# Machine learning–assisted directed evolution of plant Rubisco

**DOI:** 10.1126/sciadv.aeh3246

**Published:** 2026-10-07

**Authors:** Julie L. McDonald, Jiacheng Lin, Yunlong Zhao, Brian L. Hie, Rosemary Birch, Mary Gehring, Bryan D. Bryson, Spencer M. Whitney, Matthew D. Shoulders, Robert H. Wilson

**Affiliations:** ^1^ Department of Chemistry, Massachusetts Institute of Technology, Cambridge, MA 02139, USA.; ^2^ Department of Biology, Massachusetts Institute of Technology, Cambridge, MA 02139, USA.; ^3^ Sarafan ChEM-H, Stanford University, Stanford, CA 94305, USA.; ^4^ Department of Biochemistry, Stanford University School of Medicine, Stanford, CA 94305, USA.; ^5^ Plant Science Division, Research School of Biology, The Australian National University, Canberra, ACT 2601, Australia.; ^6^ Whitehead Institute for Biomedical Research, Cambridge, MA 02142, USA.; ^7^ Department of Biological Engineering, Massachusetts Institute of Technology, Cambridge, MA 02139, USA.; ^8^ Howard Hughes Medical Institute, Whitehead Institute for Biomedical Research, Cambridge, MA 02142, USA.; ^9^ Ragon Institute of Massachusetts General Hospital, Harvard, and MIT, Cambridge, MA 02139, USA.; ^10^ Broad Institute of MIT and Harvard, Cambridge, MA 02142, USA.; ^11^ Koch Institute for Integrative Cancer Research at MIT, Cambridge, MA 02141, USA.

## Abstract

Ribulose-1,5-bisphosphate carboxylase/oxygenase (Rubisco) is foundational to life on Earth, catalyzing carbon dioxide (CO_2_) fixation to generate biomass. However, Rubisco is a slow and inefficient enzyme that has proven challenging to engineer. We applied the structure-informed machine learning (ML) model ESM-IF1 to identify plausible amino acid sites in the large subunit of *Nicotiana tabacum* Rubisco to target for directed evolution. ML-assisted library design followed by selection in Rubisco-dependent *Escherichia coli* identified multiple enriched variants displaying improved catalytic efficiency. Several improved variants carried amino acid changes not found in the evolutionary lineage of plants, despite being assembly competent in plant chloroplasts, demonstrating that ML-assisted protein design can explore functional sequence space beyond what is observed from natural sequence diversity. Most prominently, the T391I substitution improved carboxylation rate by 29% and aerobic carboxylation efficiency by 43%. Our findings illustrate the utility of ML-assisted evolution for engineering Rubisco with improved carboxylation efficiency and potential for enhancing crop productivity.

## INTRODUCTION

Rubisco is the primary CO_2_-fixing enzyme on Earth, yet it has several shortcomings that limit carbon acquisition, particularly in land plants (*1*–*3*). Rubisco carboxylates its five-carbon substrate ribulose-1,5-bisphosphate (RuBP) slowly, necessitating its excessive production in the leaves of plants where Rubisco comprises 20 to 50% of soluble protein (*4*). Furthermore, Rubisco catalyzes RuBP oxygenation, producing 2-phosphoglycolate that C_3_ plants metabolize through photorespiration at a loss of energy, and carbon as CO_2_ (*5*). Rubisco’s catalytic limitations constrain photosynthesis in major C_3_ crops such as soybean, rice, and wheat, making the enzyme a prominent target for improving productivity (*1*).

Expanding knowledge of Rubisco’s kinetic diversity across plant and non-plant lineages has reshaped views of its adaptive potential and called into question the long-held “catalytic trade-off,” where gains in $k_{\text{cat}}^{c}$ were thought to only arise at the expense of reducing CO_2_/O_2_ specificity (*S*_C/O_) (*6*). Evidence now indicates that this apparent trade-off within vascular plant isoforms is weaker than initially expected (*6*) and may largely reflect the enzyme’s slow natural evolutionary pace and historical adaptation to its CO_2_ environment (*7*–*10*). Laboratory engineering, freed from these evolutionary constraints, has been used to push different Rubisco lineages beyond their natural limits, yielding amino acid substitutions that enhance carboxylation kinetics (*11*–*15*). Among these engineered variants are single substitutions that substantially increase $k_{\text{cat}}^{c}$ without compromising, and sometimes enhancing, *S*_C/O_, thus demonstrating that Rubisco’s catalytic landscape is more flexible than previously hypothesized (*16*). The biophysical limits of Rubisco’s carboxylase and oxygenase activities remain a key topic of research relevant to understanding the enzyme’s adaptation to the emergence of atmospheric oxygen, independent evolution of CO_2_ concentrating mechanisms in diverse branches of photosynthetic life (*17*, *18*), and reduced inhibition by oxygen observed within the red lineage of Form ID Rubisco (*19*).

Engineering of plant Rubisco was historically bottlenecked by an inability to express the enzyme recombinantly in a tractable laboratory host, owing to a dependence on multiple specialized biogenesis chaperones (*20*, *21*). Coexpression of these chaperones alongside the Rubisco large (RbcL) and small (RbcS) subunits permits holoenzyme production in *Escherichia coli* for several dicot (*21*–*25*), monocot (*26*), and nonvascular basal plants (*27*).

Expression in *E. coli* of ancestral *Solanaceae* Rubisco reconstructions yielded enzymes with improved carboxylation efficiency relative to *Nicotiana tabacum* (tobacco) Rubisco, mediated by multiple substitutions in RbcL and RbcS (*22*). More recently, directed evolution of tobacco Rubisco using random mutagenesis and Rubisco-dependent *E. coli* (RDE) screening was successful in identifying a single *Nt*RbcL residue change (Met^116^→Leu; M116L) that increased $k_{\text{cat}}^{c}$ and *S*_C/O_ (*12*). Although the M116L substitution did not improve carboxylation efficiency, the findings show that high-throughput engineering of plant Rubisco is possible and that directed evolution may eventually offer catalytic improvements to plant Rubisco similar to those already achieved with cyanobacterial (*14*), proteobacterial (*11*, *28*), and archaeal (*13*) isoforms.

Traditional directed evolution approaches face a vast sequence space that is impossible to search completely through random mutagenesis. For more efficient library design strategies, protein engineering campaigns have begun to turn to protein language models (pLMs). Such models are pretrained on large libraries of sequence and structure information, leading to models that can deliver improved protein functions, ranging from protein-protein interactions to catalytic enhancement (*29*–*39*). Related to Rubisco in particular, a supervised Gaussian process model was recently applied to predict $k_{\text{cat}}^{c}$, $K_{C}^{\text{air}}$, and *S*_C/O_ of many natural Rubisco sequences (*40*), supporting the specific application of machine learning (ML) for Rubisco engineering. However, whether pLMs can identify Rubisco substitutions that improve catalysis remains unclear.

Here, we apply pretrained, unsupervised ML models to recommend single amino acid substitutions in tobacco Rubisco with high evolutionary and structural likelihood. Although this likelihood score is a term that is only representative of evolutionary plausibility, improvements in likelihood have been observed to translate to increased substrate affinity, kinetics, catalytic activity, and thermostability (*34*, *35*), making it a suitable parameter to optimize during Rubisco evolution. We use log-likelihood scores to inform construction of a site-saturated *Nt*RbcL variant library, which proved markedly more efficient than random mutagenesis and enabled the recovery of multiple variants with higher carboxylation efficiency using an RDE selection system (*14*). Several enriched variants incorporated substitutions absent from known vascular plant Rubisco enzymes, underscoring the ability of ML-assisted evolution to explore rare sequence solutions. These substitutions included the most successful variant, *Nt*RbcL Thr^391^→Ile (T391I), which displayed a 29% faster $k_{\text{cat}}^{c}$ and 43% improvement in aerobic carboxylation efficiency ($k_{\text{cat}}^{c}/K_{C}^{\text{air}}$). Using a transgenic *Nicotiana benthamiana* line dependent on bacterial *Rhodospirillum rubrum* Rubisco (RbcM) for growth, we further confirmed that ML-recommended, catalytically improved *Nt*RbcL variants could assemble correctly in chloroplasts. Collectively, these results underscore the potential of ML-assisted Rubisco engineering to improve CO_2_ fixation catalysis and, if translated to stably transformed vascular plants, to strengthen crop photosynthesis and productivity.

## RESULTS

### Structure-informed in silico deep mutational scan (DMS) of *Nt*RbcL

We first tested whether the sequence-only model ensemble ESM-1bv (*41*, *42*), which has previously been used to inform directed evolution of other proteins (*34*), could effectively recommend beneficial substitutions in the catalytic large subunit of tobacco Rubisco (table S1). ESM-1bv generally suggested amino acid substitutions already common in vascular plant Rubisco outside *Solanaceae* (fig. S1). However, such phylogenetically widespread substitutions are not reliable indicators of kinetic improvement, given that carboxylation efficiency shows little correlation with evolutionary relatedness. That is, highly diverged land plant lineages often display near identical kinetic profiles (*43*). Furthermore, physiological (*44*) and environmental adaptation (*45*) can generate substantial kinetic variation even among closely related species. These considerations suggested to us that a sequence-only model may be insufficient to identify mutations that meaningfully enhance *Nt*RbcL catalysis.

To ground recommendations within the overall architecture and catalytic mechanism of Rubisco, we moved beyond sequence-only models to explore a protein structure-informed ML-based design strategy. ESM-IF1 (*35*, *46*), trained on 12 million predicted structures generated by AlphaFold2 (*47*), is designed to generate a protein’s amino acid sequence from an input structural backbone (*48*), meaning that it attempts to predict a set of optimized sequences best adapted to conserved folds. We used ESM-IF1 to score the log-likelihood of single amino acid substitutions at each position in *Nt*RbcL relative to wild type (Fig. 1A and table S1) using crystal structures of tobacco Rubisco in both the unactivated “open” [Protein Data Bank (PDB) ID: 1RLC] (*49*) and activated “closed” (PDB ID: 4RUB) (*50*) configurations. The closed structure contains additional backbone structural information in *Nt*RbcL due to ordering of the C terminus over the catalytic Loop 6 when Rubisco is activated and substrate bound (*51*). Rubisco in both structures is bound to 2-carboxyarabinitol-1,5-bisphosphate (CABP), a nonhydrolyzable analog mimicking the carboxylation transition state. We reasoned that supplying bound structures to ESM-IF1 would help to avoid recommendations that alter the backbone conformation in ways that preclude substrate binding (Fig. 1B).

**Fig. 1.**
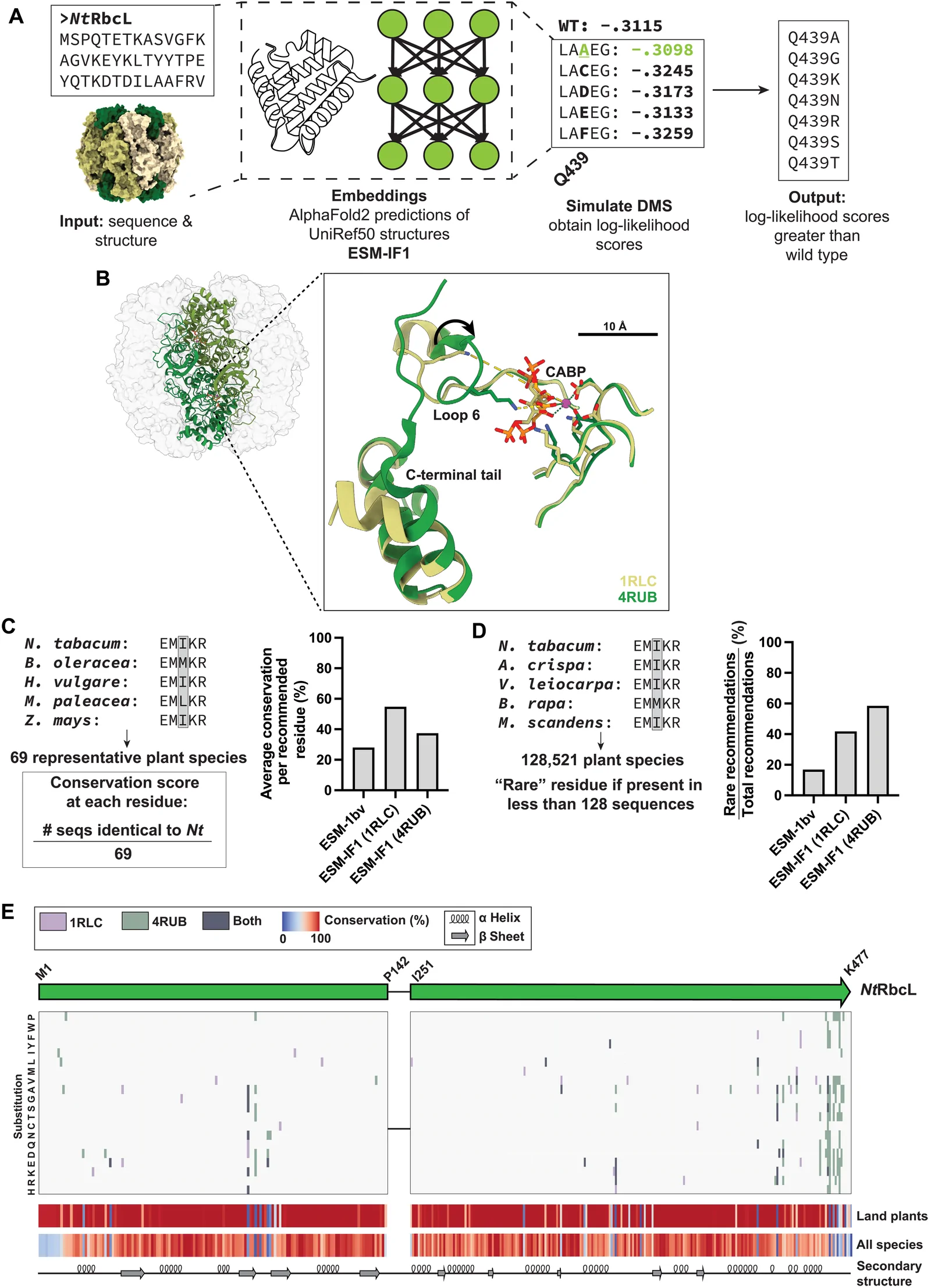
Evolution of *Nt*RbcL using ESM-IF1 in silico DMS. (**A**) ESM-IF1 inputs structural information alongside protein sequence. In silico DMS resulted in several recommended variants with log-likelihood scores greater than wild-type *Nt*RbcL. (**B**) Input structures used for ESM-IF1. PDB ID: 4RUB differs from PDB ID: 1RLC by activation (carbamylation) of the catalytic lysine, causing the closure of Loop 6 toward the bound substrate and compaction of the C terminus toward the active site. (**C**) Average conservation of amino acid loci recommended by ESM-1bv and ESM-IF1. Full species names and RbcL GenBank accession codes are listed in table S2. (**D**) Percentage of the top 24 ESM-1bv and ESM-IF1–recommended substitutions that occur rarely (<0.01% of the time) in sequenced plant Rubisco. (**E**) Location of recommended substitutions in the *Nt*RbcL sequence using different input structures. Conservation and secondary structure at each residue is shown. Approximate boundaries of the N-terminal, TIM barrel, and C-terminal domains are indicated. Sequence is not shown from residues 143 to 250 as no ML recommendations were made in this region.

In total, 202 substitutions at 49 positions across both structures displayed log-likelihood scores greater than the corresponding wild-type amino acid in *Nt*RbcL. Of these substitutions, 29 were recommended using both structural inputs and 20 overlapped with recommendations from the ESM-1bv ensemble (table S1). Recommendations from ESM-IF1 were, on average, located at more highly conserved residues than recommendations from ESM-1bv. We calculated a conservation score for each residue in *Nt*RbcL using a diverse dataset of land plant sequences (*9*), where the score is the percentage of sequences in the dataset that contain the same amino acid at a given position as *Nt*RbcL (Fig. 1C). The average conservation score of the 18 amino acid sites that comprise the 24 amino acid substitutions recommended by ESM-1bv was 28%. For consistency with the ESM-1bv predictions, we calculated the average conservation score of sites within the 24 most highly ranked ESM-IF1 recommendations. These sites had an average conservation score of 37 to 55%, depending on input structure. We also surveyed a larger dataset of ∼128,000 plant Rubisco sequences to determine how often the models recommended amino acid substitutions that are rarely observed in natural plant Rubisco sequences (Fig. 1D). Seventeen percent of substitutions recommended by ESM-1bv were found in <0.01% of plant Rubisco sequences, and all rare substitutions were recommended by only one of the six models in the ensemble. In contrast, 42 and 58% of the top 24 ESM-IF1 recommendations were rare for “open” (PDB ID: 1RLC) and “closed” (PDB ID: 4RUB) structural inputs, respectively.

Many ESM-IF1 recommendations were located in the C terminus of *Nt*RbcL (Fig. 1E), especially for the closed structure. Encouragingly, there were recommendations where congruent substitutions enhance catalytic parameters in distantly related Rubisco isoforms, such as Trp^451^→Phe in the archaeal Rubisco from *Methanococcoides burtonii* (as Tyr^471^) (*13*). No substitutions were predicted with a higher log-likelihood score than the wild type at site Phe^345^, a locus that is commonly selected for its solubility-enhancing outcome during directed evolution of related Rubisco from cyanobacteria (*14*, *52*–*54*).

### Preliminary screening and characterization of structure-informed *Nt*RbcL sequence recommendations

We sought to evaluate whether the predicted structure-based substitutions permitted Rubisco folding and function using the RDE2 bacterial screening and selection platform. RDE2 is a second-generation RDE system in which phosphoribulokinase (PRK) is expressed from an arabinose-inducible promoter, causing the accumulation of cytotoxic RuBP that is removed through Rubisco catalysis (*14*). We used a two-plasmid system that enables plant Rubisco expression in *E. coli* and is compatible with RDE2 (*12*, *24*) to screen tobacco Rubisco variants. As in prior work, the Rubisco plasmid used, pET-*Nt*LS-Rca, contains a Val^369^→Ala (V369A) substitution in *Nt*RbcL and is referred to as “wild type” henceforth. V369A does not substantially affect catalysis but does improve the enzyme’s solubility in *E. coli* (table S3). The use of an already high-solubility *Nt*RbcL variant helps to ensure selection in RDE2 on the basis of improved kinetic properties rather than merely improved solubility (*11*, *14*).

For an initial trial, three substitutions in *Nt*RbcL from 202 recommendations were manually selected. The substitution Ile^465^→Val (I465V) was chosen because of its previous selection during directed evolution of cyanobacterial Rubisco (*52*, *53*), although such selection was potentially due to solubility effects. We also chose two previously uncharacterized charge-switching substitutions, Ala^438^→Arg(A438R) and Gln^439^→Lys (Q439K), based on their proximity to one another and differences in their natural occurrence. R438 was not observed in natural sequences surveyed and K439 is found only in bacterial Rubisco (Fig. 2A), although the similarly positively charged residue R439 is widespread in plant Rubisco.

**Fig. 2.**
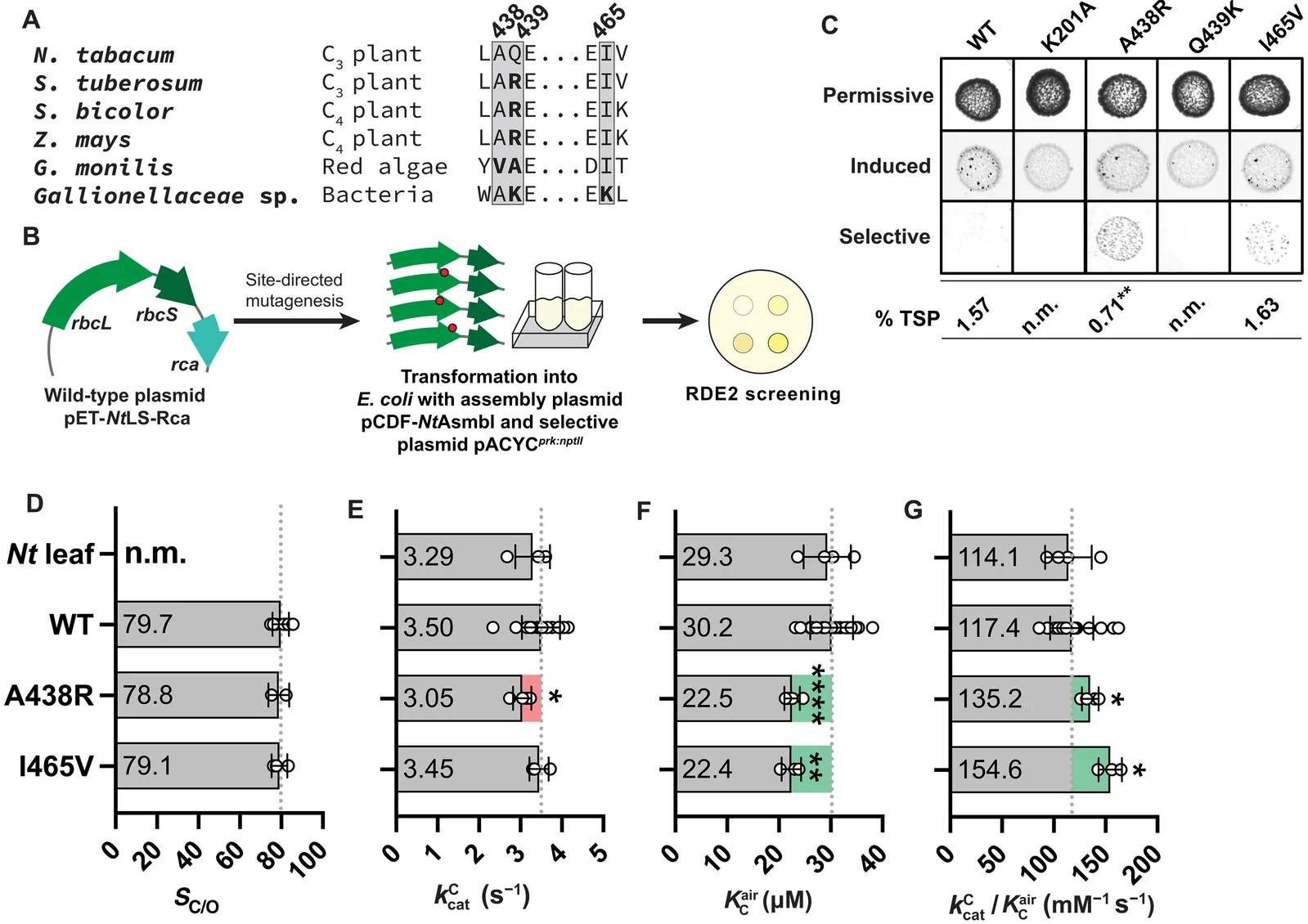
Characterization of select ESM-IF1–recommended NtRbcL variants. (**A**) Sequence alignment of Rubisco across phylogeny. ESM-IF1–recommended sites are boxed in gray and sequence differences relative to *Nt*RbcL are bolded. Full species names and RbcL GenBank accession codes are listed in table S2. (**B**) Workflow: Plasmids (pET-*Nt*LS-Rca) coding wild-type (WT) or ESM-IF1–recommended substitutions in *Nt*RbcL were transformed into RDE2 containing plasmids pACYC*^prk:nptII^* for PRK-dependent selection and pCDF-*Nt*Asmbl for assembly of the tobacco Rubisco holoenzyme. (**C**) RDE2 spot tests for comparing Rubisco-facilitated growth on permissive media (no additives), Rubisco-inducing media (0.2 mM IPTG), and Rubisco + PRK-selective media [0.15% (w/v) l-arabinose, kanamycin (400 μg/ml), and 0.2 mM IPTG]. Soluble Rubisco content in *E. coli*, quantified by [^14^C]-CABP binding (*85*), is shown as % total soluble protein (% TSP). (**D**) Specificity factor; *S*_C/O_. (**E**) Carboxylation rate; $k_{\text{cat}}^{c}$. (**F**) Affinity for CO_2_ in air; $K_{C}^{\text{air}}$. (**G**) Carboxylation efficiency in air; $k_{\text{cat}}^{c}/K_{C}^{\text{air}}$. Significance testing for (C) to (G) was performed via two-tailed, heteroscedastic *t* tests of each Rubisco variant relative to the wild type. *P ≤ 0.05; **P ≤ 0.005; ****P ≤ 0.00005. All error bars represent the SD. Replicate data for (C) to (G) are listed in table S4. Dashed lines in (D) to (G) represent the mean of wild-type measurements for each parameter.

We generated the *Nt*RbcL A438R, Q439K, and I465V variants using site-directed mutagenesis and screened them using RDE2 alongside an inactive Rubisco negative control (*Nt*RbcL^Lys201→Ala (K201A)^; Fig. 2B). After 4 days under RDE selection on strongly selective media containing 0.15% (w/v) l-arabinose in an atmosphere containing 0.5% (v/v) CO_2_ and 21% (v/v) O_2_, *E. coli* expressing the wild-type, *Nt*RbcL^Q439K^, or *Nt*RbcL^K201A^ tobacco Rubisco failed to grow. Cells producing *Nt*RbcL^A438R^ and *Nt*RbcL^I465V^ survived (Fig. 2C). Neither the *Nt*RbcL^A438R^ nor *Nt*RbcL^I465V^ mutants improved Rubisco solubility (Fig. 2C), indicating their fitness-enhancing properties were likely catalytic and motivating further characterization of these two variants.

Measurements of $k_{\text{cat}}^{c}$ using soluble protein in the *E. coli* lysate exhibited high interexperiment variability relative to *N. tabacum* leaf soluble protein controls (fig. S2). Attempts to purify Rubisco from the *E. coli* lysate using a chromatographic purification protocol optimized for Rubisco purification from leaves (*55*) proved to be low-throughput and resulted in low yields of still heavily contaminated end product (fig. S3). To simplify the purification process, we adapted a nickel-immobilized metal affinity chromatography (IMAC) purification method previously used to purify *Synechococcus* sp. PCC6301 Rubisco (*52*) by appending an N-terminal cleavable histidine-tagged ubiquitin (^His6^Ubq) to the Rubisco small subunit (*Nt*^His6^UbqRbcS; fig. S4, A and B). Coexpression of *Nt*RbcL, untagged *Nt*RbcS, and *Nt*^His6^UbqRbcS allowed for the purification of tobacco Rubisco complexes by IMAC before cleaving off the ubiquitin tag (fig. S4C) (*56*). Using this method, the isolated Rubisco contained no amino acid epitope tags, was typically >70% pure, and resulted in more reproducible measurements of $k_{\text{cat}}^{c}$ and $K_{C}^{\text{air}}$ (fig. S2).

Compared with wild-type tobacco Rubisco, the *Nt*RbcL^A438R^ and *Nt*RbcL^I465V^ variants showed no differences in *S*_C/O_ (Fig. 2D). The $k_{\text{cat}}^{c}$ of *Nt*RbcL^A438R^ was slightly decreased relative to the wild type, and the $k_{\text{cat}}^{c}$ of *Nt*RbcL^I465V^ was unchanged (Fig. 2E). However, the apparent CO_2_ affinity ($K_{C}^{\text{air}}$) of both *Nt*RbcL^A438R^ and *Nt*RbcL^I465V^ improved by ∼25% (Fig. 2F), leading to 15 and 32% improvements in their carboxylation efficiency ($k_{\text{cat}}^{c}/K_{C}^{\text{air}}$), respectively (Fig. 2G). Thus, in accordance with previous work screening plant (*12*) and bacterial (*13*, *14*) Rubisco variants, improved RDE2 selective growth could be ascribed to an improvement in catalysis.

To more deeply analyze ESM-IF1 recommendations, we generated site-saturated mutagenesis libraries at the 49 recommended *Nt*RbcL amino acid sites (table S1), along with K201 as a negative control. This library of ∼400 variants (Fig. 3A) contained at least one substitution at each target site aside from R89, which failed during insert fragment synthesis (table S5). Next-generation sequencing (NGS) coverage of the plasmid was highly variable, especially at *rbc*L (fig. S5). Excision of *rbc*L was apparent during library generation, indicating that cytotoxicity or plasmid instability from the repetitive T7 promoter and terminator sequences (fig. S4A) may have prevented construction of all possible *Nt*RbcL variants.

**Fig. 3.**
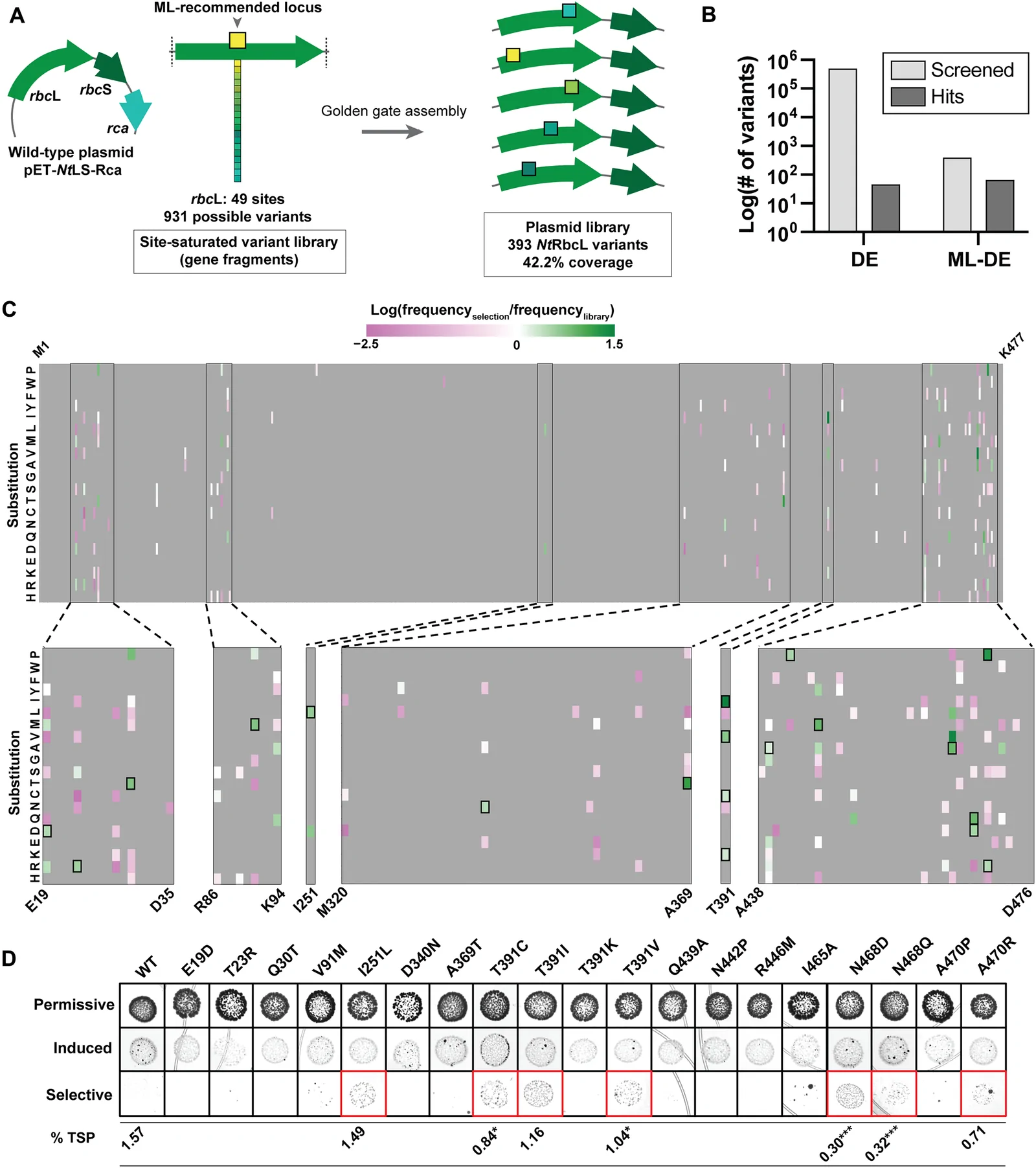
Library screening at ESM-IF1–recommended sites. (**A**) Construction of a site-saturated variant library at 49 sites by the Golden Gate assembly. (**B**) Tobacco Rubisco variants screened and isolated as “hits” from ML-assisted directed evolution (ML-DE; this work) and traditional directed evolution [DE; (*12*)]. Hits from DE screening were identified as fast-growing colonies in RDE2, whereas hits from ML-DE screening were identified through enrichment in NGS reads. (**C**) Enrichment of variants in RDE2 selection. Enrichment score was calculated as the frequency of the variant on selective media divided by the frequency of the variant in the starting library. Heatmap represents log scale enrichment scores (table S5). (**D**) RDE2 rescreening of enriched variants. Media conditions are equivalent to those in (Fig. 2C). Soluble Rubisco content in *E. coli* for each measured variant is shown. Significance is shown relative to wild-type tobacco Rubisco as analyzed via a two-tailed, heteroscedastic *t* test. *P ≤ 0.05; ***P ≤ 0.0005.

Library selection in RDE2 followed by NGS found 64 substitutions, including I465V, that were enriched following selection relative to their frequency in the starting library, whereas substitutions at the catalytically critical K201 locus were consistently depleted (Fig. 3, B and C, and table S5). The overall positive enrichment rate was 16%, 1700-fold higher compared to prior work selecting from error-prone polymerase chain reaction (PCR) libraries of *N. tabacum* Rubisco that identified 46 enriched variants from a library of ∼500,000 (*12*) (Fig. 3B). ML-assisted library preparation is therefore effective for generating small, streamlined Rubisco libraries that successfully navigate the high degree of sequence conservation present in land plant isoforms (Fig. 1E).

Of the 19 chosen Rubisco variants enriched under >0.1% arabinose selection (Fig. 3C, black boxes), seven showed improved RDE2 growth relative to the wild type that was not attributable to increased Rubisco solubility (Fig. 3D, red boxes). We additionally combined the top 5, 10, and 20 most highly enriched point mutations to test for additive benefit to RDE2 growth (fig. S6). The quintuple variant *Nt*RbcL resulted in slightly worse growth in RDE2 compared to the most highly enriched variant (*Nt*RbcL^T391I^), suggesting no apparent improvement to *Nt*RbcL function or solubility with the addition of more substitutions. The variants containing 10 and 20 substitutions failed to grow under selective conditions, indicating that these combinations of substitutions impaired Rubisco folding and/or function.

### ML-assisted engineering achieves improved carboxylation efficiency

*Nt*RbcL containing the single substitutions Ile^251^→Leu(I251L), Thr^391^→Cys(T391C), T391I, Thr^391^→Val (T391V), Asn^468^→Asp (N468D), Asn^468^→Gln (N468Q), or Ala^470^→Arg (A470R) were purified and carboxylation catalysis was measured under ambient atmospheric O_2_ [21% (v/v), equilibrated as 253 μM dissolved O_2_] to mimic the physiological conditions of a plant chloroplast (*57*) and to obtain $K_{C}^{\text{air}}$ values to model the response of leaf photosynthetic rate (*A*) under varying chloroplast CO_2_ pressures (*C*_c_) (*58*) (Table 1 and table S4). The carboxylation properties of the most strongly enriched RDE2 variant, *Nt*RbcL^T391I^, were the most improved, with a 29% higher $k_{\text{cat}}^{c}$ and 11% reduction in $K_{C}^{\text{air}}$ to give a net 43% increase in $k_{\text{cat}}^{c}/K_{C}^{\text{air}}$. *Nt*RbcL^I251L^ and *Nt*RbcL^T391V^, as well as the previously measured variants *Nt*RbcL^A438R^ and *Nt*RbcL^I465V^ (Fig. 2G), also displayed improved $k_{\text{cat}}^{c}/K_{C}^{\text{air}}$ (Fig. 4A) owing to reductions to $K_{C}^{\text{air}}$ while maintaining a similar $k_{\text{cat}}^{c}$ to the wild type. *Nt*RbcL^T391C^, *Nt*RbcL^N468D^, and *Nt*RbcL^N468Q^ displayed a similar catalytic trend of improved $K_{C}^{\text{air}}$, although overall improvement to carboxylation efficiency was not significant for these variants (Table 1). Measurements of *S*_C/O_ (Fig. 4B) revealed that none of the amino acid changes significantly affected the overall specificity of the enzyme for CO_2_ over O_2_.

**Table 1.** Kinetic characterization of *Nt*RbcL variants at 25°C. Values provided are means ± SD. Replicate data are provided in table S4. Statistical significance relative to the wild type was calculated via a two-tailed, heteroscedastic *t* test. *P ≤ 0.05; **P ≤ 0.005; ***P ≤ 0.0005; ****P ≤ 0.00005.

*Nt*RbcL	Enrichment score	$k_{\text{cat}}^{c}$ (s^−1^)	$K_{C}^{\text{air}}$ (μM)	$k_{\text{cat}}^{c}/K_{C}^{\text{air}}$ (mM^−1^ s^−1^)	*S*_C/O_
Wild-type	–	3.5 ± 0.5	30.2 ± 4.1	117 ± 21	79.7 ± 4.0
I251L	17.7	3.1 ± 0.1*	22.0 ± 1.2****	142 ± 7**	79.8 ± 3.1
T391C	8.4	3.3 ± 0.3	24.9 ± 0.7***	131 ± 9	79.2 ± 0.6
T391I	189.3	4.5 ± 0.4*	26.9 ± 1.0*	168 ± 19*	79.9 ± 3.1
T391V	2.2	3.3 ± 0.1	23.0 ± 1.4***	143 ± 12*	78.7 ± 3.3
A438R	–	3.0 ± 0.2*	22.5 ± 1.5****	135 ± 8*	78.8 ± 5.0
I465V	136.0	3.5 ± 0.2	22.4 ± 1.9**	155 ± 11*	79.1 ± 3.8
N468D	13.5	3.0 ± 0.3	20.2 ± 6.4	156 ± 32	76.7 ± 3.5
N468Q	36.1	3.1 ± 0.3	20.7 ± 0.5****	152 ± 18	80.8 ± 1.3
A470R	12.5	3.6 ± 0.3	29.9 ± 4.6	124 ± 20	77.4 ± 0.9

**Fig. 4.**
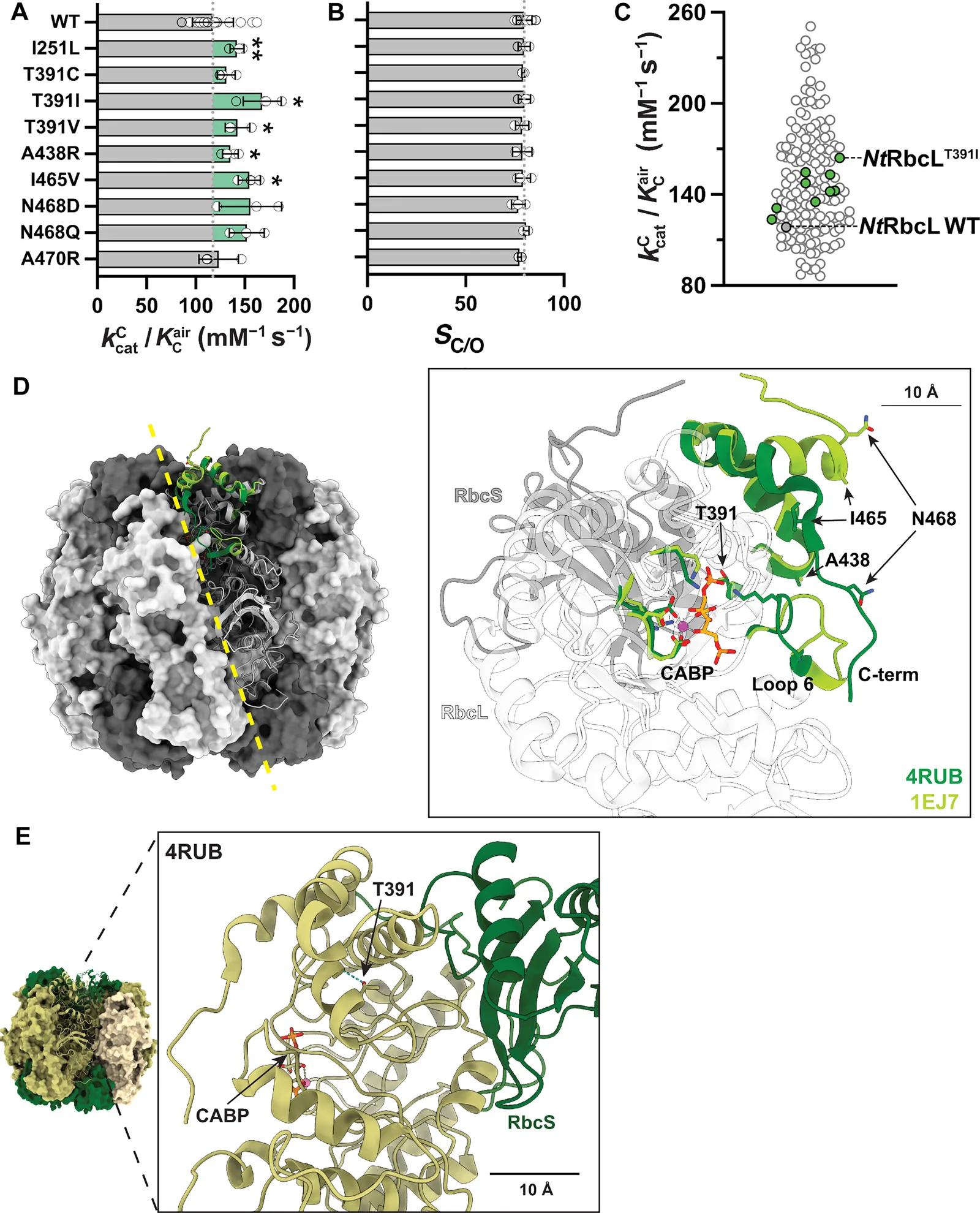
Tobacco Rubisco variants in kinetic and structural contexts. (**A**) Carboxylation efficiency in air ($k_{\text{cat}}^{c}/K_{C}^{\text{air}}$) and (**B**) CO_2_/O_2_ specificity factor (*S*_C/O_) for wild-type *Nt*RbcL and variants. Statistical significance relative to the wild type was calculated via a two-tailed, heteroscedastic *t* test. *P ≤ 0.05; **P ≤ 0.005. (**C**) Carboxylation efficiencies in air of C_3_ plants, displayed with data collected in this study and from (*59*). *Nt*RbcL variants measured in this work are shown as filled green circles, whereas wild-type *Nt*RbcL is shown as a filled gray circle. (**D**) Structural analysis of amino acids near the *Nt*RbcL active site that enhance carboxylation catalysis. PDB ID: 4RUB (*50*) (activated, closed, and CABP-bound) and 1EJ7 (*88*) (unactivated, open, and phosphate-bound). (**E**) Structural contextualization of *Nt*RbcL^T391I^ between *Nt*RbcL and *Nt*RbcS within the Rubisco hexadecamer. PDB ID: 4RUB.

Previously analyzed plant Rubisco, especially those from C_3_ plants, occupy a narrow range (*59*) of carboxylation efficiency (86 to 251 mM^−1^ s^−1^) in their natural aerobic environment [i.e., under 21% (v/v) ambient O_2_ (*9*, *59*, *60*)]. Compared with $k_{\text{cat}}^{c}/K_{C}^{\text{air}}$ data collated in a previous study (*59*), we found that our ML-assisted directed evolution approach enabled tobacco Rubisco to traverse across 30% of the C_3_ plant Rubisco catalytic landscape via single substitutions alone (Fig. 4C). As each naturally evolved amino acid substitution that occurs in RbcL is proposed to increase the enzyme’s anaerobic carboxylation efficiency by only ∼1% (*9*), the ML-based approach taken here identified functional mutations that appear to sidestep this natural constraint (for aerobic carboxylation efficiency).

Given that these beneficial substitution sites were predicted using structural information alongside protein sequence, we aimed to understand the potential impact of these substitutions on the overall structure of the holoenzyme. Several of the catalytically improved variants, specifically A438R, I465V, N468D, and N468Q, are located in the C-terminal portion of the enzyme (Fig. 4D), a highly mobile region directly involved in the catalytic mechanism, where it operates to latch Loop 6 in the closed state following RuBP binding. This process requires the breakage and formation of several hydrogen bonds, thereby contributing to the overall energetics of the reaction (*51*, *61*). I465 and N468 in particular are positioned at the terminus of the final α helix of *Nt*RbcL, which becomes more disordered during C-terminal-tail repositioning. I465 contributes to a spiral channel along with residues Phe^467^(F467) and Phe^469^ (F469), which flank N468. This channel has been proposed to funnel gas substrates to the active site (*62*) and may explain the observed improvements in $K_{C}^{\text{air}}$ for *Nt*RbcL^I465V^, and by *Nt*RbcL^N468D/Q^, through stabilization or favorable positioning of the F467 and F469 side chains within the funnel. We note that the gas funnel is not an experimentally substantiated feature of Rubisco catalysis and that both F467 and F469 form critical interactions with the assembly chaperone RbcX (*63*) that may alternatively explain their high sequence conservation and the detrimental influence of N468 mutants on Rubisco solubility (Fig. 3D).

T391 is positioned near the active site, closer to the small subunit interface than the C terminus (Fig. 4E). The hydroxyl group of the threonine side chain of residue 391 forms a hydrogen bond with the peptide backbone at Met^387^ (M387), stabilizing the α helix. This interaction is eliminated upon substitution with the nonpolar amino acids isoleucine or valine. I251 uniquely faces the internal cavity of the holoenzyme and varies as methionine, valine, or leucine at this position across related enzymes and is one of the most positively selected residues in photosynthetic RbcL (*64*). Both M251 and V251 were not present in the screened library and will require further studies to determine whether variation at this site is a catalytic switch.

Substitutions T391I and A438R may alter the active site architecture in a way that reduces the activation energy of CO_2_ addition to RuBP or increases the activation energy of O_2_ addition (*65*), which may underlie the observed improvement in $K_{C}^{\text{air}}.$ Similarly, T391I may influence structural rearrangements of the active site during catalysis to accelerate CO_2_ addition as an explanation for the marked improvement to $k_{\text{cat}}^{c}$ exhibited by this variant. Future detailed kinetic characterization of oxygen affinity ($K_{O}^{}$) and the derived $k_{\text{cat}}^{o}$, will prove useful in understanding how mutations at positions 438 and 391 influence the oxygenation properties of tobacco Rubisco.

### Improved *Nt*RbcL variants can assemble in planta

To further position the *Nt*RbcL variants within the context of Rubisco evolution, we conducted a detailed phylogenetic analysis of the amino acid identity at selected sites (fig. S7). Only the evolved residues L251 and D468 appear naturally, and only at a low frequency, in sequenced vascular plants. In comparison, I391 and V465 are generally observed only in distantly related Rubisco from diatoms and bacteria, respectively, and R438 is virtually never observed despite the catalytic benefit these residues confer to *N. tabacum* Rubisco.

Although these substitutions allow Rubisco production in a heterologous bacterial system, we wondered whether biogenesis disruption in chloroplasts may explain their absence in plants. Notably, the production of heterologous plant RbcL in leaf chloroplasts is challenged by the protracted timelines to generate a chloroplast genome (plastome)–transformed line. We therefore developed a transgenic *N. benthamiana* line *^Nb^rbc*M that is suited to rapidly screen the chloroplast assembly compatibility of RbcL via *Agrobacterium*-mediated transient expression. This *^Nb^rbc*M line was generated by plastome transformation with plasmid p^cm^trLA, previously used to enable the same replacement in *N. tabacum* cv. Petit Havana (*66*) (Fig. 5A). In *^Nb^rbc*M the native *rbc*L plastome gene is replaced with a codon-modified ^cm^*rbc*M gene coding the smaller (100 kDa) *R. rubrum* Form II Rubisco dimer (RbcL_2_) along with the *aad*A gene coding for spectinomycin resistance. Both *^Nb^rbc*M lines generated were spectinomycin resistant and required elevated CO_2_ for growth in soil, owing to the poor catalytic efficiency and oxygen sensitivity of *R. rubrum* Rubisco (fig. S8).

**Fig. 5.**
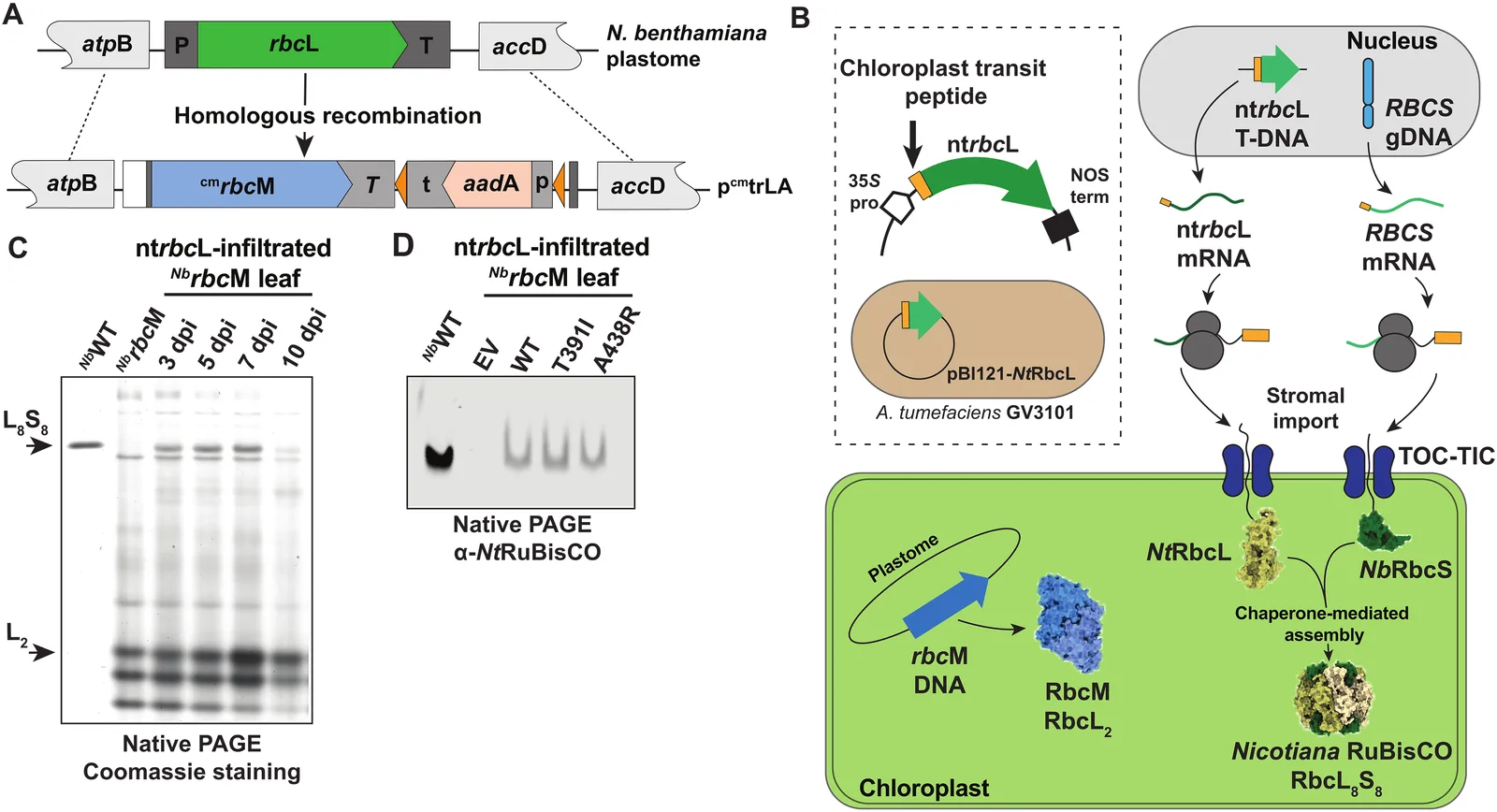
*Nt*RbcL variants fold and assemble in leaf chloroplasts. (**A**) Plastome transformation of *N. benthamiana* with plasmid p^cm^trLA directed the replacement of *rbc*L with a codon-modified *rbc*M (^cm^*rbc*M) gene and *aad*A spectinomycin resistance marker gene to generate independent *^Nb^rbc*M lines (fig. S8). P, 5′UTR and the first 42 bp of the *rbc*L coding sequence; T, 285 bp of 3′UTR; *T*, 222 bp of *psb*A 3′UTR; p, 16*S* rDNA *rrn* promoter and 5′UTR; t, *rps*16 3′UTR (*66*). Orange triangles represent loxP sites for marker excision, if desired. (**B**) Schematic of the transient RbcL expression and RbcL_8_S_8_ production in the *^Nb^rbc*M chloroplasts via agroinfiltration of plasmid pBI121-*Nt*RbcL. Shown are how the endogenous nuclear *RBCS* mRNA copies and *rbc*L transgene are translated in the cytosol as precursor peptides coding equivalent N-terminal chloroplast transit peptides that facilitate their import into the chloroplast stroma for assembly into the Rubisco holoenzyme. (**C**) Native PAGE of the leaf soluble protein isolated from pBI121-*Nt*RbcL agroinfiltrated leaves 3 to 10 dpi. Infiltrated leaf protein was loaded at fivefold excess relative to the wild-type sampled leaf area to visualize production of ∼520-kDa tobacco L_8_S_8_ Rubisco and the 100-kDa *R. rubrum* L_2_ Rubisco via Coomassie staining. (**D**) Native PAGE Western blot against tobacco Rubisco for native, wild-type *N. benthamiana* (*^Nb^*WT), and *^Nb^rbc*M expressing wild-type and variant *Nt*RbcL. EV, empty vector sampled from an equivalent leaf area.

As seen in equivalent *R. rubrum* Rubisco-producing *N. tabacum* (*66*) and *Solanum tuberosum* (*67*) (potato) genotypes, the *^Nb^rbc*M lines retained RbcS and Rubisco chaperone production from their nuclear-encoded genes (Fig. 5B). Agroinfiltration of plasmids coding *N. tabacum* RbcL and the 57 amino acid N-terminal chloroplast-transit peptide from RbcS thus allows for the transient production of RbcL_8_S_8_ complexes within the chloroplasts of ^*Nb*^*rbc*M, as confirmed by native polyacrylamide gel electrophoresis (PAGE) screens that showed tobacco holoenzyme production between 3–7 days postinfiltration (dpi; Fig. 5C). Infiltration of equivalent constructs coding the I391 and R438 substitutions yielded detectable levels of assembled Rubisco at 5 dpi (Fig. 5D). Thus, despite these residues being extremely rare in natural plant RuBisCO lineages (fig. S7 and table S1), their production is supported in a chloroplast environment.

Leaf photosynthesis rates in C_3_ plants can be simulated by integrating in vitro–derived Rubisco catalytic parameters into the established biochemical Farquhar, von Caemmerer, and Berry model (*12*, *13*, *68*–*71*). In chloroplasts producing equivalent amounts of Rubisco (20 μmol catalytic sites m^−2^), the *Nt*RbcL^T391I^ mutant is predicted to support the greatest gain in Rubisco activity-limited photosynthetic potential. *Nt*RbcL^I465V^ and *Nt*RbcL^N468D^ variants provided the next highest rates after *Nt*RbcL^T391I^ (Fig. 6). As the *S*_C/O_ of each variant remained equivalent to wild-type Rubisco (Table 1), the electron transport–limited rates of photosynthesis (when light-dependent RuBP production becomes most limiting) are predicted by the model to arise at lower *C*_c_ values. Nevertheless, each mutant is predicted to support between 15 to 25% faster CO_2_ assimilation rates under current atmospheric CO_2_ levels of 420 parts per million (ppm), which correspond to in planta *C*_c_ pressures between ∼250 and 320 μbar.

**Fig. 6.**
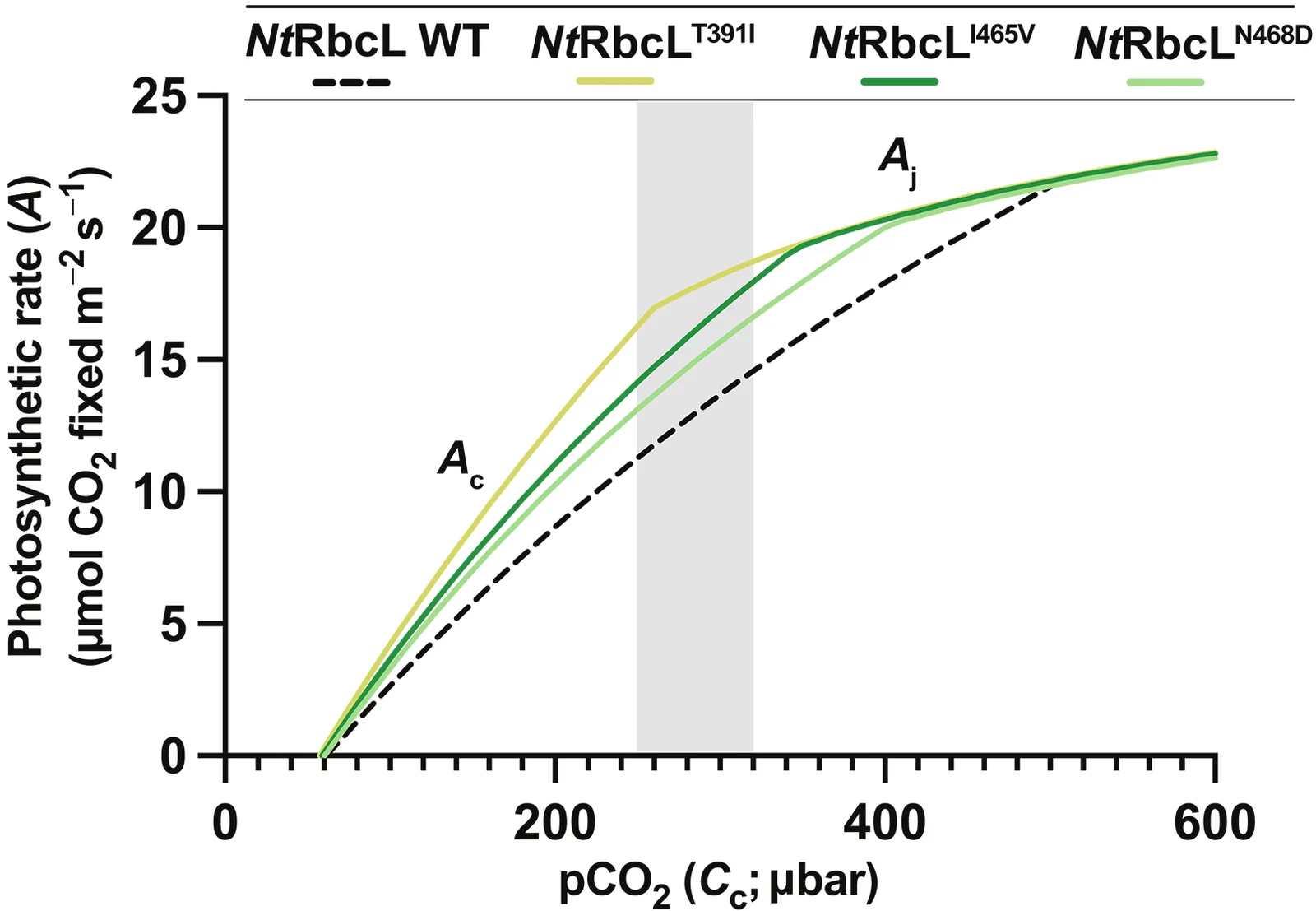
*Nt*RbcL variants support higher modeled rates of photosynthesis in C_3_ leaves. Modeled Rubisco activity limited (*A*_c_) and electron transport–limited (*A*_j_) photosynthetic assimilation rates at 25°C in C_3_ leaves expressing variant or wild-type tobacco Rubisco using the kinetic parameters listed in Table 1. Photosynthetic simulations are as described by (eqs. S1 and S2). pCO_2_ (*x* axis) represents the partial pressure of dissolved CO_2_ in a C_3_ chloroplast, and the shaded region represents the typical CO_2_ concentration of a C_3_ chloroplast (250 to 320 μbar) under current atmospheric CO_2_ (420 ppm).

## DISCUSSION

In this work, we used ML to inform the directed evolution of tobacco Rubisco, yielding variants with improved $k_{\text{cat}}^{c}$ and $K_{C}^{\text{air}}$ in vitro. By using the structure-informed pLM ESM-IF1 to recommend mutagenesis sites for directed evolution libraries, we were able to focus sequence space exploration toward residue changes more likely to be functionally relevant compared to random mutagenesis (Fig. 3B), avoiding the numerous deleterious mutations that would have been sampled using error-prone PCR. This approach allowed for substantial minimization of the starting mutational library to reduce the cost and difficulty of generating and maintaining large sequence libraries. In addition, the use of ML-assisted site-saturation mutagenesis allows for amino acid substitutions that require dinucleotide substitutions in the parental codon that would be under-sampled using single round error-prone PCR. This approach may explain why A438R (a GCT to CGT codon substitution) was not identified from nt*rbc*L variants generated from random mutagenesis libraries (*12*).

ESM-IF1 was less conservative in mutational predictions compared to the sequence-only ML ensemble ESM-1bv, due to the high level of primary amino acid sequence conservation present in land plant Rubisco (fig. S1). Models, such as ESM-IF1, trained on structural databases potentially avoid such constraint by focusing attention on biophysical principles of the Rubisco structure rather than existing evolutionary trajectories.

Although successful at predicting function-enhancing amino acid sites in *Nt*RbcL, ESM-IF1 did not always predict the highest-performing substitution at each site. For example, *Nt*RbcL^T391I^ was not initially predicted. Only Thr^391^→Arg (T391R) was recommended by ESM-IF1 and that substitution was not enriched following selection in RDE2. On the other hand, some substitutions that enhanced catalysis, such as I465V, were directly predicted, as was the substitution M116L, a substitution that improves $k_{\text{cat}}^{c}$ by 30% previously identified during *N. tabacum* Rubisco directed evolution driven by error-prone PCR (*12*). Site-saturation mutagenesis at key ML-recommended sites, in combination with in vivo selection, may therefore be a productive strategy for plant Rubisco engineering.

Structure-informed ML through ESM-IF1 suggested substitutions in tobacco Rubisco that are rare in, or absent from, other vascular plant Rubisco sequences (fig. S7). Through ML-assisted directed evolution in *E. coli*, we found that tobacco Rubisco enzymes containing these substitutions were not only catalytically enhanced (Table 1) but were also assembly competent in the native chloroplast environment (Fig. 5D). Functional substitutions at these sites may be under-sampled by methodologies primarily reliant on extant sequence information (*40*), including sequence-only ESM models.

A promising future direction for ML-assisted Rubisco engineering comes from supervised ML models, which input kinetic data alongside structure and sequence information to predict beneficial amino acid substitutions. These models could provide a further advantage when designing Rubisco sequence libraries as they can also encode known information about key protein-protein interactions in planta. Such encoding is essential as many changes recommended by the unsupervised models we tested, such as Arg^89^→Pro (R89P) and Lys^94^→Glu (K94E), are known to interrupt interaction with Rubisco activase (Rca) (*72*–*74*), which can cause severe reductions in plant photosynthetic efficiency (*75*, *76*). These and other critical protein-protein interactions therefore do not seem to be captured by general unsupervised pLMs and must be considered when testing substitutions recommended by such models. Supervised models have already been used to predict extant Rubisco kinetics (*40*), and our results here indicate they may also be useful when used to generate variant predictions or entirely redesigned sequences.

The best-performing variant we isolated, *Nt*RbcL^T391I^, displayed an ∼43% higher carboxylation efficiency in air than wild-type *Nt*RbcL, mostly owing to its faster carboxylation rate. This rate increase was achieved without compromising *S*_C/O_, an increasingly familiar outcome of carefully analyzed Rubisco directed evolution studies (*11*–*14*, *28*). Other selected amino acid substitutions at position 391 in NtRbcL (T391C and T391V) did not improve $k_{\text{cat}}^{c}$ but did benefit the enzyme’s affinity for CO_2_ via reductions in $K_{C}^{\text{air}}$ relative to wild-type *Nt*RbcL, suggesting that this locus may be an important modulator of catalysis that is unexplored in plant Rubisco sequence space. Notably, the original substitution predicted by ESM-IF1 (T391R) was not enriched by selection in RDE2. The remaining improved variants displayed enhanced $K_{C}^{\text{air}}$ leading to significant improvement to carboxylation efficiency for the I251L, A438R, and I465V substitutions.

Large-scale experimental surveys of Rubisco catalysis from vascular plants demonstrate substantial variation in each catalytic parameter across natural isoforms (*77*), which encompass the variation exhibited by single-site substitutions characterized in this study. These surveys broadly found *S*_C/O_ to correlate positively with $K_{C}^{\text{air}}$ and negatively with carboxylation efficiency in air ($k_{\text{cat}}^{c}/K_{C}^{\text{air}}$) (*43*). However, up to threefold variation in $k_{\text{cat}}^{c}$ and $K_{C}^{\text{air}}$ can be observed among enzymes with relatively similar *S*_C/O_ values. The improvements presented in this study, alongside those observed from ancestral reconstructions of Rubisco from the *Solanaceae* family (*22*), show that engineered enhancements are accessible and have the potential to result in improved leaf photosynthetic rates at physiological CO_2_ concentrations.

In conclusion, we used ML-assisted directed evolution to enhance the carboxylation kinetics of tobacco Rubisco through improvements to both carboxylation rate and affinity for CO_2_ in air. It remains possible that factors such as reductions in chloroplast Rubisco content (*78*), increased xylulose-1,5-bisphosphate (XuBP) production (*79*), and reduced activation state (*72*, *78*) caused by these substitutions in planta may have prevented their evolutionary adoption in plants, especially given their predicted benefits to C_3_ photosynthesis (Fig. 6). Construction and full analysis of stable plastome-transformed *N. tabacum* lines expressing these mutant *rbc*L alleles will be necessary to analyze and understand these potential limitations, as well as how the kinetic changes affect plant photosynthesis and productivity.

This work supports continued efforts to engineer plant Rubisco, including further rounds of ML-facilitated evolution of tobacco and other crop Rubisco and expanding the explored sequence space to include RbcS due to its influential role on substrate specificity (*80*). Ultimately, successful translation of improved Rubisco kinetics in planta has the potential to improve crop photosynthesis toward enhancing crop yields to meet rising global food needs.

## MATERIALS AND METHODS

### ESM model implementation

ESM-1bv recommendations were generated as described previously (*34*, *35*), outputting 24 amino acid substitutions. In silico DMS using ESM-IF1 was performed as described previously (*35*), and scores are available in table S1. All relevant code for ESM recommendations and figure generation is available at https://github.com/julielmcdonald/RubiscoMLDE.

### Sequence alignment and conservation analysis

We used the Streptophyta sequence dataset curated by Bouvier *et al.* (*9*) to calculate land plant conservation scores relative to *Nt*RbcL. A total of 69 sequences in this dataset were aligned using MAFFT (*81*), and the conservation score at each residue was calculated as the percentage of sequences in the alignment that contained the same amino acid at a given position relative to *Nt*RbcL.

To calculate the conservation score for all species, as shown in Fig. 1E, Jackhmmer (*82*) was used to search for sequences with homology to *Nt*RbcL in the UniProtKB/Swiss-Prot database, yielding 740 representative sequences for conservation analysis. Sequences coding for Rubisco-like proteins were manually removed, resulting in an alignment of 679 protein sequences from bacteria, algae, and plants. Conservation scores were then calculated as above.

To determine which recommendations from ESM were rare in plant Rubisco (table S1) and for alignment of winning sites (fig. S7), RbcL sequences from varying taxonomies were extracted from GenBank and aligned using MAFFT. Full analyses, including code used to survey residues at each locus, are available at https://github.com/julielmcdonald/RubiscoMLDE.

### Variant library generation and selection

For the ESM-IF1 site-saturated variant library, gene fragments containing saturated mutants at selected sites were obtained from Twist Biosciences and were amplified using the primers gg-1 and gg-2. The plasmid pET-*Nt*LS-Rca was amplified using the primers gg-3 and gg-4 (table S7). Fragments were PCR purified (Omega) and assembled using the Golden Gate Assembly via *Bsa*I-HF (New England Biolabs) cutting and T4 ligase (New England Biolabs) ligation overnight. The product of the Golden Gate reaction was purified (Omega), 100 ng was electroporated into NEB Stable *E. coli* (New England Biolabs), and the outgrowth was plated onto 10 LB agar plates containing ampicillin (100 μg/ml). After 2 days of growth at 30°C, ∼400 colonies were scraped from the plates and the plasmid pool was purified (Omega).

For selection of both libraries, 150 ng of the library was electroporated into BL21 Star *E. coli* (Invitrogen) containing the plasmids pACYC*^prk:nptII^* and pCDF-*Nt*Asmbl. The cells were grown at 37°C for 1.5 hours before plating on selective agar plates containing ampicillin (100 μg/ml), spectinomycin (100 μg/ml), chloramphenicol (35 μg/ml), tetrazolium chloride (TTC; 50 μg/ml), 0.2 mM isopropyl-β-d-thiogalactopyranoside (IPTG), kanamycin (400 μg/ml), and increasing concentrations of l-arabinose [0.05 to 0.2% (w/v)]. Plates were incubated for 2 days at 25°C in air supplemented with 0.5% (v/v) CO_2_. All colonies from each selection condition were scraped, and the plasmid pool was purified for NGS. Variant nt*rbc*L selected for RDE rescreening were generated using the Q5 site-directed mutagenesis kit (New England Biolabs) or were synthesized by GenScript Inc.

### Next-generation sequencing

Plasmids from the input library and all selection conditions were linearized, sequenced, and analyzed as described previously (*11*). We used the Genome Analysis Toolkit (GATK) (*83*) to identify single-nucleotide polymorphisms (SNPs) in pET-*Nt*LS-Rca. Mutations at 0.10% or a higher frequency (determined using a read quality score of 30) in all samples, including the input library, are detailed in table S5.

### RDE spot testing

Colonies of pET-*Nt*LS-Rca, pCDF-*Nt*Asmbl, and pACYC*^prk:nptII^* cotransformed into BL21 Star *E. coli* (Invitrogen) were grown on LB plates containing ampicillin (100 μg/ml), spectinomycin (100 μg/ml), and chloramphenicol (35 μg/ml; LB-ACS). A colony was used to inoculate 1 ml of LB-ACS, which was then incubated at 37°C and 180 rpm to an optical density at 600 nm (OD_600_) of ∼0.2. The resulting culture was diluted with LB to an equivalent OD_600_ of 1 × 10^−4^, and 20 μl was spotted onto an LB-ACS plate containing TTC (50 μg/ml), 0.2 mM IPTG, kanamycin (400 μg/ml), and incremental concentrations [0 to 0.2% (w/v)] of l-arabinose. The plates were incubated at 25°C in air supplemented with 0.4% (v/v) CO_2_. Growth was monitored over 8 days.

### Histidine-ubiquitin-tagged Rubisco purification

Calcium-competent BL21 Star *E. coli* were cotransformed with pET-*Nt*LS-Rca (containing the wild type or mutated nt*rbc*L), pCDF-*Nt*Asmbl, and pACYC-*Nt* ^His6^Ubq-RbcS. A colony from this transformation was picked and grown in 25 ml of LB-ACS overnight. Twenty milliliters of overnight culture was used to inoculate 1 liter of LB-ACS. This culture was grown at 37°C at 180 rpm to OD_600_ = 0.8 (∼2.5 hours), at which point protein expression was induced via addition of IPTG to a final concentration of 0.5 mM and the temperature was reduced to 23°C. Growth continued for 18 hours, and then cells were pelleted by centrifugation for 15 min at 4000*g* at 4°C. Cell pellets were snap frozen in liquid N_2_ for storage at −80°C.

For Rubisco purification, the stored cell pellets were resuspended in 20 ml of ice-cold lysis buffer [30 mM EPPS-NaOH at pH 8.0, 150 mM NaCl, 10 mM imidazole (Alfa Aesar), 1 mM phenylmethylsulfonyl fluoride (PMSF; Amresco), 2 mM dithiothreitol (DTT; Sigma-Aldrich), and 1 μl of DNase (Sigma-Aldrich)] and then lysed using a needle-tipped sonicator (30% amplitude with six 20 s on/off cycles) on ice. Following centrifugation (20,000*g* for 30 min at 4°C), the soluble protein was filtered through a 0.22 μm filter before IMAC purification using a 5 ml HisTrap excel column (Cytiva) equilibrated with lysis buffer. After loading soluble lysate, the column was washed with 100 ml of lysis buffer containing 60 mM imidazole. Rubisco proteins were then eluted with 12 ml of lysis buffer containing 300 mM imidazole. For ubiquitin tag cleavage, 400 μl of 80% (v/v) glycerol, 40 μl of 1.5 M β-mercaptoethanol (Sigma-Aldrich), and 150 μl of USP2 protease (2 mg/ml) was added to the elution. The resulting solution was incubated at 30°C for 30 min to initiate cleavage of the ubiquitin tag and then dialyzed at 4°C overnight against dialysis buffer [10 mM EPPS-NaOH at pH 8.0, 150 mM NaCl, 1 mM EDTA, and 5% (v/v) glycerol]. The USP2 protease was purified as previously described (*56*).

The dialyzed protein was subjected to IMAC using a 5 ml HisTrap excel column equilibrated with dialysis buffer to remove the histidine-tagged USP2 protease and any uncleaved Rubisco. Untagged, pure Rubisco was collected in the flow-through and was concentrated using an Amicon Ultra Centrifugal Filter (Sigma-Aldrich) with a 100 kDa molecular weight cutoff. Protein was buffer exchanged into storage buffer (30 mM EPPS-NaOH at pH 8.0, 150 mM NaCl, 1 mM EDTA, and 1 mM DTT) and was snap frozen for future use.

### Untagged Rubisco purification from *E. coli*

BL21 Star *E. coli* cultures were grown and induced as described above, with the omission of the plasmid pACYC-*Nt* ^His6^Ubq-RbcS and chloramphenicol in the growth medium. Cells were resuspended in lysis buffer (30 mM Tris-HCl at pH 8.0, 5 mM DTT, 2 mM PMSF, and 1 μl of DNase) and lysis proceeded as above, and then the lysate was applied to an ENrich Q 5 x 50 anion exchange column (Bio-Rad) using a Bio-Rad NGC fast protein liquid chromatography system. Rubisco was eluted from anion exchange using a salt gradient of 0 to 600 mM NaCl. Rubisco fractions eluted at ∼210 mM NaCl were pooled, concentrated, and loaded onto an ENrich SEC 650 10 x 300 column (Bio-Rad) for size exclusion chromatography. Rubisco was eluted using 30 mM Tris-HCl at pH 8.0, 50 mM NaCl, and 10 mM MgCl_2_. Fractions containing Rubisco (fig. S3) were pooled and then snap frozen for future use.

### Rubisco content and kinetic characterization

Purified Rubisco were used to determine *S*_C/O_ using the [^3^H]-RuBP consumption assay (*84*). ^14^CO_2_ fixation assays to measure $k_{\text{cat}}^{c}$ and $K_{C}^{\text{air}}$ were undertaken in reactions equilibrated with CO_2_-free air at 25°C containing five incremental ^14^CO_2_ concentrations between 0 and 300 μM, as previously described (*44*, *85*). Briefly, the purified *Nt*RbcL protein was diluted to 2 μM in assay buffer (100 mM EPPS-NaOH at pH 8.0, 25 mM MgCl_2_, and 1 mM EDTA) and activated for at least 10 min with 10 mM NaH^14^CO_3_ before diluting 50-fold into a 0.5-ml reaction containing 1.3 μM RuBP. After 3 min, the reaction was quenched with 50% (v/v) formic acid, unincorporated ^14^C was evaporated overnight, and the fixed ^14^C was counted using a Beckman LS 6500 scintillation counter. Counts were normalized to the number of Rubisco active sites in the purified protein sample, quantified by [^14^C]-CABP binding, as previously described (*85*, *86*).

For measurement of Rubisco content in soluble protein of cellular lysates, 200 μl of stationary-phase BL21 Star *E. coli* cotransformed with pET-*Nt*LS-Rca and pCDF-*Nt*Asmbl was used as a starter culture to inoculate a 10-ml culture of LB-ACS in a 50-ml baffled flask. Following growth at 37°C at 180 rpm to an OD_600_ of 0.8 (∼2.5 hours), Rubisco expression was induced with 0.5 mM IPTG and the cells were grown at 23°C for 18 hours before centrifuging for 10 min at 4000*g* at 4°C and snap-freezing the cell pellets in liquid N_2_ for storage at −80°C. Cell pellets were resuspended in ice-cold lysis buffer (30 mM EPPS-NaOH at pH 8.0, 150 mM NaCl, 25 mM MgCl_2_, 10 mM NaHCO_3_, 1 mM PMSF, 2 mM DTT, and 1 μl of DNase) and lysed using a needle-tipped sonicator (20% amplitude with two 20 s on/off cycles) on ice. Following centrifugation (20,000*g*, 15 min, 4°C), the soluble protein was used for ^14^C-CABP binding, as previously described (*85*, *86*), and protein quantification via a Pierce Bradford Protein Assay Kit against bovine serum albumin. Rubisco concentration as a percentage (w/w) of total cellular soluble protein assumed a molecular mass of 67 kDa for each *Nt*RbcL and RbcS pair. When prepared for kinetics assays, *E. coli* lysates were treated analogously except with the exclusion of 10 mM NaHCO_3_ from the lysis buffer. Twenty microliters of the lysate was activated for at least 10 min with the addition of NaH^14^CO_3_ to 10 mM and then was diluted 1:1 in assay buffer and used for kinetics measurements as described above.

### SDS-PAGE, Western blotting, and native PAGE

SDS-PAGE was completed using NuPAGE Bis-Tris Mini Protein Gels, 4 to 12% (Thermo Fisher Scientific), following the manufacturer’s instructions (200 V for 30 min). Native PAGE was completed using Novex Value Tris-Glycine Mini Protein Gels, 4 to 12% (Thermo Fisher Scientific), following the manufacturer’s instructions (120 V for 2.5 hours at 4°C). Sample loading was normalized to soluble protein content, with 1 μg of protein loaded for Coomassie staining and 0.5 μg of protein loaded for immunoblotting. For immunoblotting, separated proteins were transferred onto a nitrocellulose membrane (Bio-Rad) using a Trans-Blot Turbo Transfer System (Bio-Rad), following the manufacturer’s specifications. Immunoblotting was performed as previously described, using a polyclonal tobacco antibody that reacts with both *Nt*RbcL and *Nt*RbcS (*87*).

### Generation and growth of *^Nb^rbc*M

Replacement of the plastome *rbc*L gene in *N. benthamiana* was achieved via transformation of the plasmid p^cm^trLA (table S6; GenBank AY827488). In p^cm^trLA, 1126 and 1176 base pairs (bp) of flanking *N. benthamiana* plastome sequence directs the homologous replacement of *rbc*L and most of its 3′ untranslated region (3′UTR) with the codon-modified *R. rubrum rbc*M gene (^cm^*rbc*M) and an inversely oriented *aad*A selective marker gene (*66*). Pure p^cm^trLA plasmid was coated onto tungsten and transformed into the abaxial side of 10 sterile *N. benthamiana* leaves using a helium accelerator biolistic gun, as previously described (*66*) with rupture discs (1100 psi). After 2 days, the leaves were dissected into ∼1 cm^2^ disks and embedded into selective 1× Murashige and Skoog (MS)/phytagel media containing spectinomycin (0.5 μg/ml), 1% (w/v) sucrose, thiamine (1 μg/ml; Sigma-Aldrich), α-naphthaleneacetic acid (0.1 μg/ml; Sigma-Aldrich), and 6-benzylaminopurine (1 μg/ml; Sigma-Aldrich). The leaf sections were incubated at 25°C in air containing 2.5% (v/v) CO_2_.

Three spectinomycin-resistant green calli were dissected after 7 weeks and then transferred to plates of fresh selective MS/phytagel media. The tissue was regenerated for 12 weeks, and then ∼0.1 cm^2^ tissue samples were regenerated again on selective MS/phytagel media. After 17 weeks, the stems of ∼1 cm high plants were cut diagonally, and the plants were propagated into 0.8 liters of polypropylene pots containing 0.15 liters of MS/phytagel media supplemented with 0.05% (w/v) sucrose and spectinomycin (0.25 mg/ml) at 25°C in 2.5% (v/v) CO_2_-enriched air. Four to 6 weeks later, following root development, the plants were transferred to plastic containers filled with a 2:1 dry volume ratio mix of vermiculite/seed-raising soil mix and a teaspoon of osmocote and were covered with 0.8 liter polypropylene pots for 6 days to maintain humidity. After 2 weeks, the plants were transferred to 2 liter pots of soil and were grown to reproductive maturity in a growth chamber at 25/20°C (16-hour light/8-hour dark), 150 to 450 μmol photons m^−2^ s^−1^ illumination, and 2% (v/v) CO_2_-enriched air. Progress toward homoplasmicity was monitored by native PAGE analysis.

### Agroinfiltration of *^Nb^rbc*M

Before germination, seeds were frozen at −80°C overnight and then were sterilized by incubation in 1% (v/v) bleach for 10 min. Seeds were washed five times with ultrapure water and then were spread onto 1× MS/phytagel media for germination. Seeds were incubated at 25°C in a growth chamber equilibrated with air supplemented with 2.0% (v/v) CO_2_ and 25 to 100 μmol photons m^−2^ s^−1^ illumination for ∼2 weeks. Seedlings were then transferred to soil and were grown for approximately three more weeks until suitable for infiltration.

*Agrobacterium tumefaciens* GV3101 was used for transient expression of *Nt*RbcL in *^Nb^rbc*M. Plasmids pBI121-*Nt*RbcL and pBI-p19 (containing the gene silencing suppressor P19) were individually transformed into electrocompetent *A. tumefaciens*. Colonies from these strains were grown in LB containing kanamycin (100 μg/ml) and rifampicin (50 μg/ml) to an OD_600_ of 0.6 (∼24 hours). One milliliter of each pBI121-*Nt*RbcL–containing culture was added to 0.5 ml of pBI-p19–containing culture and then pelleted by centrifugation at 15,000*g* for 1 min at 24°C. Cells were resuspended in 3 ml of infiltration buffer (10 mM MES-NaOH at pH 5.5 and 10 mM MgSO_4_) and were injected into the leaves of ∼5-week-old *^Nb^rbc*M plants using a syringe. Plants were grown for 6 days after infiltration with watering on day 3, and then 0.5 cm^2^ leaf disks were punched and snap frozen for future use. Leaf samples removed from −80°C were immediately resuspended in 1 ml of lysis buffer [30 mM EPPS-NaOH at pH 8.0, 150 mM NaCl, 25 mM MgCl_2_, 1 mM PMSF, 2 mM DTT, 1 μl of DNase, and 1% (w/v) polyvinylpolypyrrolidone (PVPP; Sigma-Aldrich)] and were mechanically lysed. After centrifugation at 20,000*g* for 15 min at 4°C to remove insoluble debris, the supernatant was prepared for SDS-PAGE analysis as described above.

### Modeling of photosynthetic rates

Photosynthetic *A*-*C*_c_ curves for C_3_-plants were modeled according to Farquhar *et al.* (*58*) using in vitro measures of Rubisco *S*_C/O_, $k_{\text{cat}}^{c}$, and $K_{C}^{\text{air}}$ (Table 1) (*1*, *58*), a leaf RuBisCO content of 20 μmol RbcL sites m^−2^, a CO_2_ solubility constant of 0.0334 M bar^−1^, an electron transport rate (*J*) of 120 μmol m^−2^ s^−1^, a nonphotorespiratory CO_2_ release rate (*R*_*d*_) of 1 μmol m^−2^ s^−1^, and a stromal O_2_ concentration of 253 μM.

## Supplementary Materials

### The PDF file includes:

Legends for tables S1, S4, S5 and S8 Tables S2, S3, S6 and S7 Equations S1 and S2 Figs. S1 to S8 References

## References

[1] M. Gionfriddo, T. Rhodes, S. M. Whitney, Perspectives on improving crop Rubisco by directed evolution. Semin. Cell Dev. Biol. 155, 37–47 (2024). 10.1016/j.semcdb.2023.04.003 37085353

[2] R. E. Sharwood, Engineering chloroplasts to improve Rubisco catalysis: Prospects for translating improvements into food and fiber crops. New Phytol. 213, 494–510 (2017). 10.1111/nph.14351 27935049

[3] N. Prywes, N. R. Phillips, O. T. Tuck, L. E. Valentin-Alvarado, D. F. Savage, Rubisco function, evolution, and engineering. Annu. Rev. Biochem. 92, 385–410 (2023). 10.1146/annurev-biochem-040320-101244 37127263

[4] E. Carmo-Silva, J. C. Scales, P. J. Madgwick, M. A. J. Parry, Optimizing Rubisco and its regulation for greater resource use efficiency. Plant Cell Environ. 38, 1817–1832 (2014). 10.1111/pce.12425 25123951

[5] B. J. Walker, A. VanLoocke, C. J. Bernacchi, D. R. Ort, The costs of photorespiration to food production now and in the future. Annu. Rev. Plant Biol. 67, 107–129 (2016). 10.1146/annurev-arplant-043015-111709 26865340

[6] G. G. B. Tcherkez, G. D. Farquhar, T. J. Andrews, Despite slow catalysis and confused substrate specificity, all ribulose bisphosphate carboxylases may be nearly perfectly optimized. Proc. Natl. Acad. Sci. U.S.A. 103, 7246–7251 (2006). 10.1073/pnas.0600605103 PMC1464328 16641091

[7] J. Galmés, M. V. Kapralov, P. J. Andralojc, M. À. Conesa, A. J. Keys, M. A. J. Parry, J. Flexas, Expanding knowledge of the Rubisco kinetics variability in plant species: Environmental and evolutionary trends. Plant Cell Environ. 37, 1989–2001 (2014). 10.1111/pce.12335 24689692

[8] P. L. Cummins, B. Kannappan, J. E. Gready, Directions for optimization of photosynthetic carbon fixation: Rubisco's efficiency may not be so constrained after all. Front. Plant Sci. 9, 183 (2018). 10.3389/fpls.2018.00183 PMC5838012 29545812

[9] J. W. Bouvier, D. M. Emms, S. Kelly, Rubisco is evolving for improved catalytic efficiency and CO_2_ assimilation in plants. Proc. Natl. Acad. Sci. U.S.A. 121, e2321050121 (2024). 10.1073/pnas.2321050121 PMC10945770 38442173

[10] J. W. Bouvier, D. M. Emms, T. Rhodes, J. S. Bolton, A. Brasnett, A. Eddershaw, J. R. Nielsen, A. Unitt, S. M. Whitney, S. Kelly, Rubisco adaptation is more limited by phylogenetic constraint than by catalytic trade-off. Mol. Biol. Evol. 38, 2880–2896 (2021). 10.1093/molbev/msab079 PMC8233502 33739416

[11] J. L. McDonald, N. P. Shapiro, A. A. Mengiste, S. Kaines, S. M. Whitney, R. H. Wilson, M. D. Shoulders, *In vivo* directed evolution of an ultrafast Rubisco from a semianaerobic environment imparts oxygen resistance. Proc. Natl. Acad. Sci. U.S.A. 122, e2505083122 (2025). 10.1073/pnas.2505083122 PMC12260525 40587785

[12] M. Gionfriddo, R. Birch, T. Rhodes, S. Buck, T. Skinner, I. Andersson, S. Whitney, Laboratory evolution of Rubisco solubility and catalytic switches to enhance plant productivity. Nat. Plants 11, 1939–1950 (2025). 10.1038/s41477-025-02093-8 40926002

[13] R. H. Wilson, H. Alonso, S. M. Whitney, Evolving *Methanococcoides burtonii* archaeal Rubisco for improved photosynthesis and plant growth. Sci. Rep. 6, 22284 (2016). 10.1038/srep22284 PMC4772096 26926260

[14] R. H. Wilson, E. Martin-Avila, C. Conlan, S. M. Whitney, An improved *Escherichia coli* screen for rubisco identifies a protein–protein interface that can enhance CO_2_-fixation kinetics. J. Biol. Chem. 293, 18–27 (2018). 10.1074/jbc.M117.810861 PMC5766918 28986448

[15] N. Prywes, N. R. Phillips, L. M. Oltrogge, S. Lindner, L. J. Taylor-Kearney, Y.-C. C. Tsai, B. de Pins, A. E. Cowan, H. A. Chang, R. Z. Wang, L. N. Hall, D. Bellieny-Rabelo, H. M. Nisonoff, R. F. Weissman, A. I. Flamholz, D. Ding, A. Y. Bhatt, O. Mueller-Cajar, P. M. Shih, R. Milo, D. F. Savage, A map of the rubisco biochemical landscape. Nature 638, 823–828 (2025). 10.1038/s41586-024-08455-0 PMC11839469 39843747

[16] G. Bainbridge, P. Madgwick, S. Parmar, R. Mitchell, M. Paul, J. Pitts, A. J. Keys, M. A. J. Parry, Engineering Rubisco to change its catalytic properties. J. Exp. Bot. 46, 1269–1276 (1995).

[17] M. R. Badger, R. E. Sharwood, Rubisco, the imperfect winner: It’s all about the base. J. Exp. Bot. 74, 562–580 (2023). 10.1093/jxb/erac458 36412307

[18] L. Schulz, J. Zarzycki, W. Steinchen, G. K. A. Hochberg, T. J. Erb, Layered entrenchment maintains essentiality in the evolution of Form I Rubisco complexes. EMBO J. 44, 269–280 (2025). 10.1038/s44318-024-00311-1 PMC11696622 39558108

[19] Z. G. Oh, B. Askey, L. H. Gunn, Red Rubiscos and opportunities for engineering green plants. J. Exp. Bot. 74, 520–542 (2023). 10.1093/jxb/erac349 PMC9833100 36055563

[20] H. Aigner, R. H. Wilson, A. Bracher, L. Calisse, J. Y. Bhat, F. U. Hartl, M. Hayer-Hartl, Plant rubisco assembly in *E. coli* with five chloroplast chaperones including BSD2. Science 358, 1272–1278 (2017). 10.1126/science.aap9221 29217567

[21] R. H. Wilson, G. Thieulin-Pardo, F. U. Hartl, M. Hayer-Hartl, Improved recombinant expression and purification of functional plant rubisco. FEBS Lett. 593, 611–621 (2019). 10.1002/1873-3468.13352 PMC6593764 30815863

[22] M. T. Lin, H. Salihovic, F. K. Clark, M. R. Hanson, Improving the efficiency of Rubisco by resurrecting its ancestors in the family *Solanaceae*. Sci. Adv. 8, eabm6871 (2022). 10.1126/sciadv.abm6871 PMC9012466 35427154

[23] M. T. Lin, W. D. Stone, V. Chaudhari, M. R. Hanson, Small subunits can determine enzyme kinetics of tobacco rubisco expressed in *Escherichia coli*. Nat. Plants 6, 1289–1299 (2020). 10.1038/s41477-020-00761-5 32929197

[24] S. Buck, T. Rhodes, M. Gionfriddo, T. Skinner, D. Yuan, R. Birch, M. V. Kapralov, S. M. Whitney, *Escherichia coli* expressing chloroplast chaperones as a proxy to test heterologous Rubisco production in leaves. J. Exp. Bot. 74, 664–676 (2023). 10.1093/jxb/erac435 36322613

[25] Z. Cai, L. Zhao, K. Ma, G. Liu, J. Zhang, F. Gong, W. Pei, L. Xiao, Y. Li, Replacement of large subunit N terminus enabled biogenesis of different plant Rubiscos in *E. coli*. Plant Biotech. J. 23, 3382–3391 (2025). 10.1111/pbi.70162 PMC12310878 40443069

[26] J. Archer, M. Kathpalia, B. U. Lee, S. Li, T. Wang, Effects of chaperone selectivity on the assembly of plant Rubisco orthologs in *E. coli*. J. Exp. Bot. 76, 2809–2820 (2025). 10.1093/jxb/eraf140 40163664

[27] Z. G. Oh, T. A. Robison, D. H. Loh, W. S. L. Ang, J. Z. Y. Ng, F.-W. Li, L. H. Gunn, Unique biogenesis and kinetics of hornwort Rubiscos revealed by synthetic biology systems. Mol. Plant 17, 1833–1849 (2024). 10.1016/j.molp.2024.10.013 39491367

[28] Y. Zhou, S. Whitney, Directed evolution of an improved rubisco; *In vitro* analyses to decipher fact from fiction. Int. J. Mol. Sci. 20, e5019 (2019). 10.3390/ijms20205019 PMC6834295 31658746

[29] P. Kouba, P. Kohout, F. Haddadi, A. Bushuiev, R. Samusevich, J. Sedlar, J. Damborsky, T. Pluskal, J. Sivic, S. Mazurenko, Machine learning-guided protein engineering. ACS Catal. 13, 13863–13895 (2023). 10.1021/acscatal.3c02743 PMC10629210 37942269

[30] S. Mazurenko, Z. Prokop, J. Damborsky, Machine learning in enzyme engineering. ACS Catal. 10, 1210–1223 (2019).

[31] Y. Qiu, G.-W. Wei, Artificial intelligence-aided protein engineering: From topological data analysis to deep protein language models. Brief. Bioinform. 24, bbad289 (2023). 10.1093/bib/bbad289 PMC10516362 37580175

[32] E. Simon, K. Swanson, J. Zou, Language models for biological research: A primer. Nat. Methods 21, 1422–1429 (2024). 10.1038/s41592-024-02354-y 39122951

[33] J. Yang, F.-Z. Li, F. H. Arnold, Opportunities and challenges for machine learning-assisted enzyme engineering. ACS Cent. Sci. 10, 226–241 (2024). 10.1021/acscentsci.3c01275 PMC10906252 38435522

[34] B. L. Hie, V. R. Shanker, D. Xu, T. U. J. Bruun, P. A. Weidenbacher, S. Tang, W. Wu, J. E. Pak, P. S. Kim, Efficient evolution of human antibodies from general protein language models. Nat. Biotechnol. 42, 275–283 (2024). 10.1038/s41587-023-01763-2 PMC10869273 37095349

[35] V. R. Shanker, T. U. J. Bruun, B. L. Hie, P. S. Kim, Unsupervised evolution of protein and antibody complexes with a structure-informed language model. Science 385, 46–53 (2024). 10.1126/science.adk8946 PMC11616794 38963838

[36] N. Thomas, D. Belanger, C. Xu, H. Lee, K. Hirano, K. Iwai, V. Polic, K. D. Nyberg, K. G. Hoff, L. Frenz, C. A. Emrich, J. W. Kim, M. Chavarha, A. Ramanan, J. J. Agresti, L. J. Colwell, Engineering highly active nuclease enzymes with machine learning and high-throughput screening. Cell Syst. 16, 101236 (2025). 10.1016/j.cels.2025.101236 40081373

[37] B. J. Wittmann, Y. Yue, F. H. Arnold, Informed training set design enables efficient machine learning-assisted directed protein evolution. Cell Syst. 12, 1026–1045.e7 (2021). 10.1016/j.cels.2021.07.008 34416172

[38] Z. Lin, H. Akin, R. Rao, B. Hie, Z. Zhu, W. Lu, N. Smetanin, R. Verkuil, O. Kabeli, Y. Shmueli, A. D. S. Costa, M. Fazel-Zarandi, T. Sercu, S. Candido, A. Rives, Evolutionary-scale prediction of atomic-level protein structure with a language model. Science 379, 1123–1130 (2023). 10.1126/science.ade2574 36927031

[39] K. Jiang, Z. Yan, M. D. Bernardo, S. R. Sgrizzi, L. Villiger, A. Kayabolen, B. J. Kim, J. K. Carscadden, M. Hiraizumi, H. Nishimasu, J. S. Gootenberg, O. O. Abudayyeh, Rapid *in silico* directed evolution by a protein language model with EVOLVEpro. Science 387, eadr6006 (2025). 10.1126/science.adr6006 39571002

[40] W. A. Iqbal, A. Lisitsa, M. V. Kapralov, Predicting plant Rubisco kinetics from RbcL sequence data using machine learning. J. Exp. Bot. 74, 638–650 (2023). 10.1093/jxb/erac368 PMC9833099 36094849

[41] J. Meier, R. Rao, R. Verkuil, J. Liu, T. Sercu, A. Rives, Language models enable zero-shot prediction of the effects of mutations on protein function. Adv. Neural Inf. Proces. Syst. 34, 29287–29303 (2021).

[42] A. Rives, J. Meier, T. Sercu, S. Goyal, Z. Lin, J. Liu, D. Guo, M. Ott, C. L. Zitnick, J. Ma, R. Fergus, Biological structure and function emerge from scaling unsupervised learning to 250 million protein sequences. Proc. Natl. Acad. Sci. U.S.A. 118, e2016239118 (2021). 10.1073/pnas.2016239118 PMC8053943 33876751

[43] D. J. Orr, A. Alcântara, M. V. Kapralov, P. J. Andralojc, E. Carmo-Silva, M. A. J. Parry, Surveying Rubisco diversity and temperature response to improve crop photosynthetic efficiency. Plant Physiol. 172, 707–717 (2016). 10.1104/pp.16.00750 PMC5047088 27342312

[44] R. E. Sharwood, O. Ghannoum, M. V. Kapralov, L. H. Gunn, S. M. Whitney, Temperature responses of Rubisco from *Paniceae* grasses provide opportunities for improving C_3_ photosynthesis. Nat. Plants 2, 16186 (2016). 10.1038/nplants.2016.186 27892943

[45] J. Galmés, P. J. Andralojc, M. V. Kapralov, J. Flexas, A. J. Keys, A. Molins, M. A. J. Parry, M. À. Conesa, Environmentally driven evolution of Rubisco and improved photosynthesis and growth within the C_3_ genus *Limonium* (*P. lumbaginaceae*). New Phytol. 203, 989–999 (2014). 10.1111/nph.12858 24861241

[46] C. Hsu, R. Verkuil, J. Liu, Z. Lin, B. Hie, T. Sercu, A. Lerer, A. Rives, in *International Conference on Machine Learning* (PMLR, 2022), pp. 8946–8970.

[47] J. Jumper, R. Evans, A. Pritzel, T. Green, M. Figurnov, O. Ronneberger, K. Tunyasuvunakool, R. Bates, A. Žídek, A. Potapenko, A. Bridgland, C. Meyer, S. A. A. Kohl, A. J. Ballard, A. Cowie, B. Romera-Paredes, S. Nikolov, R. Jain, J. Adler, T. Back, S. Petersen, D. Reiman, E. Clancy, M. Zielinski, M. Steinegger, M. Pacholska, T. Berghammer, S. Bodenstein, D. Silver, O. Vinyals, A. W. Senior, K. Kavukcuoglu, P. Kohli, D. Hassabis, Highly accurate protein structure prediction with AlphaFold. Nature 596, 583–589 (2021). 10.1038/s41586-021-03819-2 PMC8371605 34265844

[48] C. Hsu, H. Nisonoff, C. Fannjiang, J. Listgarten, Learning protein fitness models from evolutionary and assay-labeled data. Nat. Biotech. 40, 1114–1122 (2022). 10.1038/s41587-021-01146-5 35039677

[49] K. Y. J. Zhang, D. Cascio, D. Eisenberg, Crystal structure of the unactivated ribulose 1, 5-bisphosphate carboxylase/oxygenase complexed with a transition state analog, 2-carboxy-D-arabinitol 1, 5-bisphosphate. Protein Sci. 3, 64–69 (1994). 10.1002/pro.5560030109 PMC2142487 8142899

[50] S. W. Suh, D. Cascio, M. S. Chapman, D. Eisenberg, A crystal form of ribulose-1,5-bisphosphate carboxylase/oxygenase from *Nicotiana tabacum* in the activated state. J. Mol. Biol. 197, 363–365 (1987). 10.1016/0022-2836(87)90129-x 3681999

[51] T. C. Taylor, I. Andersson, Structural transitions during activation and ligand binding in hexadecameric Rubisco inferred from the crystal structure of the activated unliganded spinach enzyme. Nat. Struct. Mol. Biol. 3, 95–101 (1996). 10.1038/nsb0196-95 8548461

[52] O. Mueller-Cajar, S. M. Whitney, Evolving improved *Synechococcus* Rubisco functional expression in *Escherichia coli*. Biochem. J. 414, 205–214 (2008). 10.1042/BJ20080668 18484948

[53] P. Durão, H. Aigner, P. Nagy, O. Mueller-Cajar, F. U. Hartl, M. Hayer-Hartl, Opposing effects of folding and assembly chaperones on evolvability of Rubisco. Nat. Chem. Biol. 11, 148–155 (2015). 10.1038/nchembio.1715 25558973

[54] M. R. Parikh, D. N. Greene, K. K. Woods, I. Matsumura, Directed evolution of RuBisCO hypermorphs through genetic selection in engineered *E. coli*. Protein Eng. Des. Sel. 19, 113–119 (2006). 10.1093/protein/gzj010 PMC2012944 16423843

[55] J. Amaral, A. K. M. Lobo, E. Carmo-Silva, D. J. Orr, “Purification of Rubisco from leaves” in *Photosynthesis: Methods and Protocols*, S. Covshoff, Ed. (Springer US, 2024), pp. 417–426. 10.1007/978-1-0716-3790-6_22 38649584

[56] R. T. Baker, A. M. Catanzariti, Y. Karunasekara, T. A. Soboleva, R. Sharwood, S. Whitney, P. G. Board, Using deubiquitylating enzymes as research tools. Methods Enzymol. 398, 540–554 (2005). 10.1016/S0076-6879(05)98044-0 16275357

[57] H. M. Steiger, E. Beck, R. Beck, Oxygen concentration in isolated chloroplasts during photosynthesis. Plant Physiol. 60, 903–906 (1977). 10.1104/pp.60.6.903 PMC542743 16660210

[58] G. D. Farquhar, S. von Caemmerer, J. A. Berry, A biochemical model of photosynthetic CO_2_ assimilation in leaves of C_3_ species. Planta 149, 78–90 (1980). 10.1007/BF00386231 24306196

[59] A. I. Flamholz, N. Prywes, U. Moran, D. Davidi, Y. M. Bar-On, L. M. Oltrogge, R. Alves, D. Savage, R. Milo, Revisiting trade-offs between rubisco kinetic parameters. Biochemistry 58, 3365–3376 (2019). 10.1021/acs.biochem.9b00237 PMC6686151 31259528

[60] Y. Savir, E. Noor, R. Milo, T. Tlusty, Cross-species analysis traces adaptation of rubisco toward optimality in a low-dimensional landscape. Proc. Natl. Acad. Sci. U.S.A. 107, 3475–3480 (2010). 10.1073/pnas.0911663107 PMC2840432 20142476

[61] S. Satagopan, R. J. Spreitzer, Substitutions at the Asp-473 latch residue of *Chlamydomonas* Ribulose bisphosphate carboxylase/oxygenase cause decreases in carboxylation efficiency and CO_2_/O_2_ specificity. J. Biol. Chem. 279, 14240–14244 (2004). 10.1074/jbc.M313215200 14734540

[62] S. Gutteridge, J. Newman, C. Herrmann, D. Rhoades, The crystal structures of Rubisco and opportunities for manipulating photosynthesis. J. Exp. Bot. 46, 1261–1267 (1995).

[63] A. Bracher, A. Starling-Windhof, F. U. Hartl, M. Hayer-Hartl, Crystal structure of a chaperone-bound assembly intermediate of form I Rubisco. Nat. Struct. Mol. Biol. 18, 875–880 (2011). 10.1038/nsmb.2090 21765418

[64] M. V. Kapralov, D. A. Filatov, Widespread positive selection in the photosynthetic Rubisco enzyme. BMC Evol. Biol. 7, 73 (2007). 10.1186/1471-2148-7-73 PMC1884142 17498284

[65] B. Stec, Structural mechanism of RuBisCO activation by carbamylation of the active site lysine. Proc. Natl. Acad. Sci. U.S.A. 109, 18785–18790 (2012). 10.1073/pnas.1210754109 PMC3503183 23112176

[66] S. M. Whitney, R. E. Sharwood, Construction of a tobacco master line to improve Rubisco engineering in chloroplasts. J. Exp. Bot. 59, 1909–1921 (2008). 10.1093/jxb/erm311 18250079

[67] T. Manning, R. Birch, T. Stevenson, G. Nugent, S. Whitney, Bacterial Form II Rubisco can support wild-type growth and productivity in *Solanum tuberosum* cv. Desiree (potato) under elevated CO_2_. PNAS Nexus 2, pgac305 (2023). 10.1093/pnasnexus/pgac305 PMC9896143 36743474

[68] Y. Zhou, L. H. Gunn, R. Birch, I. Andersson, S. M. Whitney, Grafting *Rhodobacter sphaeroides* with red algae Rubisco to accelerate catalysis and plant growth. Nat. Plants 9, 978–986 (2023). 10.1038/s41477-023-01436-7 37291398

[69] S. M. Whitney, S. von Caemmerer, G. S. Hudson, T. J. Andrews, Directed mutation of the Rubisco large subunit of tobacco influences photorespiration and growth. Plant Physiol. 121, 579–588 (1999). 10.1104/pp.121.2.579 PMC59421 10517850

[70] M. T. Lin, A. Occhialini, P. J. Andralojc, M. A. J. Parry, M. R. Hanson, A faster Rubisco with potential to increase photosynthesis in crops. Nature 513, 547–550 (2014). 10.1038/nature13776 PMC4176977 25231869

[71] A. Occhialini, M. T. Lin, P. J. Andralojc, M. R. Hanson, M. A. J. Parry, Transgenic tobacco plants with improved cyanobacterial Rubisco expression but no extra assembly factors grow at near wild-type rates if provided with elevated CO_2_. Plant J. 85, 148–160 (2016). 10.1111/tpj.13098 PMC4718753 26662726

[72] J. Ng, Z. Guo, O. Mueller-Cajar, Rubisco activase requires residues in the large subunit N terminus to remodel inhibited plant Rubisco. J. Biol. Chem. 295, 16427–16435 (2020). 10.1074/jbc.RA120.015759 PMC7705312 32948656

[73] E. M. Larson, C. M. O’Brien, G. Zhu, R. J. Spreitzer, A. R. Portis, Specificity for activase is changed by a Pro-89 to Arg substitution in the large subunit of ribulose-1,5-bisphosphate carboxylase/oxygenase. J. Biol. Chem. 272, 17033–17037 (1997). 10.1074/jbc.272.27.17033 9202018

[74] C. M. Ott, B. D. Smith, A. R. Portis Jr., R. J. Spreitzer, Activase region on chloroplast ribulose-1,5-bisphosphate carboxylase/oxygenase: Nonconservative substitution in the large subunit alters species specificity of protein interaction. J. Biol. Chem. 275, 26241–26244 (2000). 10.1074/jbc.M004580200 10858441

[75] L. H. Gunn, E. Martin Avila, R. Birch, S. M. Whitney, The dependency of red Rubisco on its cognate activase for enhancing plant photosynthesis and growth. Proc. Natl. Acad. Sci. U.S.A. 117, 25890–25896 (2020). 10.1073/pnas.2011641117 PMC7568259 32989135

[76] S. M. Whitney, R. Birch, C. Kelso, J. L. Beck, M. V. Kapralov, Improving recombinant Rubisco biogenesis, plant photosynthesis and growth by coexpressing its ancillary RAF1 chaperone. Proc. Natl. Acad. Sci. U.S.A. 112, 3564–3569 (2015). 10.1073/pnas.1420536112 PMC4371954 25733857

[77] A. Prins, D. J. Orr, P. J. Andralojc, M. P. Reynolds, E. Carmo-Silva, M. A. J. Parry, Rubisco catalytic properties of wild and domesticated relatives provide scope for improving wheat photosynthesis. J. Exp. Bot. 67, 1827–1838 (2016). 10.1093/jxb/erv574 PMC4783365 26798025

[78] M. T. Lin, D. J. Orr, D. Worrall, M. A. J. Parry, E. Carmo-Silva, M. R. Hanson, A procedure to introduce point mutations into the Rubisco large subunit gene in wild-type plants. Plant J. 106, 876–887 (2021). 10.1111/tpj.15196 33576096

[79] R. Flachmann, G. Zhu, R. G. Jensen, H. J. Bohnert, Mutations in the small subunit of ribulose-1,5-bisphosphate carboxylase/oxygenase increase the formation of the misfire product xylulose-1,5-bisphosphate. Plant Physiol. 114, 131–136 (1997). 10.1104/pp.114.1.131 PMC158286 9159945

[80] L. Schulz, Z. Guo, J. Zarzycki, W. Steinchen, J. M. Schuller, T. Heimerl, S. Prinz, O. Mueller-Cajar, T. J. Erb, G. K. A. Hochberg, Evolution of increased complexity and specificity at the dawn of form I Rubiscos. Science 378, 155–160 (2022). 10.1126/science.abq1416 36227987

[81] K. Katoh, D. M. Standley, MAFFT multiple sequence alignment software version 7: Improvements in performance and usability. Mol. Biol. Evol. 30, 772–780 (2013). 10.1093/molbev/mst010 PMC3603318 23329690

[82] L. S. Johnson, S. R. Eddy, E. Portugaly, Hidden Markov model speed heuristic and iterative HMM search procedure. BMC Bioinformatics 11, 431 (2010). 10.1186/1471-2105-11-431 PMC2931519 20718988

[83] G. A. Van der Auwera, B. D. O’Connor, *Genomics in the Cloud: Using Docker, GATK, and WDL in Terra* (O’Reilly Media Inc., 2020), pp. 496.

[84] H. J. Kane, J. Viil, B. Entsch, K. Paul, M. K. Morell, T. J. Andrews, An improved method for measuring the CO_2_/O_2_ specificity of Ribulose bisphosphate Carboxylase-Oxygenase. Funct. Plant Biol. 21, 449 (1994).

[85] D. S. Kubien, C. M. Brown, H. J. Kane, Quantifying the amount and activity of Rubisco in leaves. Methods Mol. Biol. 684, 349–362 (2011). 10.1007/978-1-60761-925-3_27 20960142

[86] S. M. Whitney, R. E. Sharwood, “Plastid transformation for Rubisco engineering and protocols for assessing expression” in *Chloroplast Biotechnology: Methods and Protocols*, Methods in Molecular Biology (Humana Press, 2014), pp. 245–262. 10.1007/978-1-62703-995-6_15 24599858

[87] S. M. Whitney, H. J. Kane, R. L. Houtz, R. E. Sharwood, Rubisco oligomers composed of linked small and large subunits assemble in tobacco plastids and have higher affinities for CO_2_ and O_2_. Plant Physiol. 149, 1887–1895 (2009). 10.1104/pp.109.135210 PMC2663749 19233903

[88] A. P. Duff, T. J. Andrews, P. M. G. Curmi, The transition between the open and closed states of rubisco is triggered by the inter-phosphate distance of the bound bisphosphate. J. Mol. Biol. 298, 903–916 (2000). 10.1006/jmbi.2000.3724 10801357

[89] E. C. Meng, T. D. Goddard, E. F. Pettersen, G. S. Couch, Z. J. Pearson, J. H. Morris, T. E. Ferrin, UCSF ChimeraX: Tools for structure building and analysis. Protein Sci. 32, e4792 (2023). 10.1002/pro.4792 PMC10588335 37774136

[90] R. E. Sharwood, O. Ghannoum, S. M. Whitney, Prospects for improving CO_2_ fixation in C_3_-crops through understanding C_4_-Rubisco biogenesis and catalytic diversity. Curr. Opin. Plant Biol. 31, 135–142 (2016). 10.1016/j.pbi.2016.04.002 27131319

[91] M. L. Matsumoto, K. C. Dong, C. Yu, L. Phu, X. Gao, R. N. Hannoush, S. G. Hymowitz, D. S. Kirkpatrick, V. M. Dixit, R. F. Kelley, Engineering and structural characterization of a linear polyubiquitin-specific antibody. J. Mol. Biol. 418, 134–144 (2012). 10.1016/j.jmb.2011.12.053 22227388

