# Supplementary material for: Machine learning–assisted directed evolution of plant Rubisco
Source: Sci Adv. 2026 Oct 7;12(41):eaeh3246. doi: 10.1126/sciadv.aeh3246 (PMC13644767; doi:10.1126/sciadv.aeh3246)
Supplement: Supplementary file 1 [file sciadv.aeh3246_sm.pdf]

Science Advances
AAAS

# Supplementary Materials for

## Machine learning–assisted directed evolution of plant Rubisco

Julie L. McDonald *et al.*

Corresponding author: Matthew D. Shoulders, mshoulde@mit.edu; Robert H. Wilson, rhwilson@mit.edu

*Sci. Adv.* **12**, eaeh3246 (2026)
DOI: 10.1126/sciadv.aeh3246

**The PDF file includes:**

Legends for tables S1, S4, S5 and S8
Tables S2, S3, S6 and S7
Equations S1 and S2
Figs. S1 to S8
References

**Other Supplementary Material for this manuscript includes the following:**

Tables S1, S4, S5 and S8

## SUPPLEMENTARY TABLES

**Table S1.** ESM-1bv *Nt*RbcL amino acid substitutions, ESM-IF1 *Nt*RbcL amino acid substitutions, and conservation scores across the *Nt*RbcL sequence (see Excel file)**.**

**Table S2.** Select RbcL sequences used for alignment.

| **Species** | **RbcL GenBank ID** |
|---|---|
| *Nicotiana tabacum* | NP_054507.1 |
| *Solanum tuberosum* | YP_635647.1 |
| *Datura stramonium* | YP_006503799.1 |
| *Glycine max* | YP_538747.1 |
| *Triticum aestivum* | NP_001392016.1 |
| *Oryza sativa* | YP_654221.1 |
| *Sorghum bicolor* | YP_009654104.1 |
| *Zea mays* | NP_043033.1 |
| *Saccharum officinarum* | YP_009389579.1 |
| *Amborella trichopoda* | NP_904107.1 |
| *Brassica oleracea* | YP_009564583.1 |
| *Hordeum vulgare* | YP_009649021.1 |
| *Marchantia paleacea* | NP_039306.1 |
| *Ardisia crispa* | YP_010714941.1 |
| *Ventilago leiocarpa* | YP_010028422.1 |
| *Brassica rapa* | XP_033129022.1 |
| *Mikania scandens* | UIS42758.1 |
| *Griffithsia monilis* | ABU53651.1 |
| *Gallionellaceae sp.* | ALP32085.1 |

**Table S3.** Solubility and kinetic characterization of $Nt\text{RbcL}^{A369V}$. Values provided are means ± standard deviation. N ≥ 3, replicate data is provided in table S4. Statistical significance relative to wild-type was calculated via a two-tailed, heteroscedastic *t*-test. ***, *p* ≤ .0005. % TSP; % total soluble protein.

| ***Nt*RbcL** | **% TSP** | $k_{cat}^{C}$ **(s$^{-1}$)** | $K_{C}^{air}$ **(μM)** | $k_{cat}^{C}/K_{C}^{air}$**(mM$^{-1}$ s$^{-1}$)** | $S_{C/O}$ |
|---|---|---|---|---|---|
| A369 | 1.57 ± 0.7 | 3.50 ± 0.5 | 30.2 ± 4.1 | 117.4 ± 20.9 | 79.7 ± 4.0 |
| V369 | 0.23 ± .1*** | 3.49 ± 1.0 | 25.3 ± 5.2 | 137.2 ± 15.4 | 82.0 ± 4.2 |

**Table S4.** Kinetic characterization of Rubisco variants (see Excel file)**.**

**Table S5.** Characterization of input plasmid library and selected variant frequencies (see Excel file)**.**

**Table S6.** Plasmids used in this study.

| Plasmid | Proteins encoded (GenBank ID) | Source |
|---|---|---|
| pET-*Nt*LS-Rca | *Nt*RbcL (P00876.2), *Nt*RbcS (P69249.1), *Nt*Rca (Q40460.1) | (*24*) |
| pCDF-*Nt*Asmbl | *Nt*Raf1 (XP_016475347.1), *Nt*Raf2 (XP_016516208.1), *Nt*RbcX (XP_016509860.1), *Nt*BSD2 (AYR18863.1), *Nt*Cpn60α (XP_016509320.1), *Nt*Cpn60β (XP_016490465.2), *Nt*Cpn20 (XP_016432480.1) | (*24*) |
| pACYC$^{prk:nptII}$ | PRK (WP_011242879.1), NptII (WP_000572405.1) | (*14*) |
| pACYC-*Nt* $^{His6}$Ubq-RbcS | $^{His6}$Ubq (AAV27297.1), *Nt*RbcS (P69249.1) | This work |
| p$^{cm}$trLA | RbcM (AAV98336.1), AadA (AAV98337.1) | (*87*) |
| pBI121-*Nt*RbcL | *tpNt*RbcL (P00876.2) | This work |
| pBI-p19 | P19 (NP_062901.1) | This work |

**Table S7.** Primers used in this study.

| Primer | Sequence |
|---|---|
| R89P | atgctaccgcatcgagcctgttgttggagaaaaag |
| V466K | cttgtgaagtatggaaagagatcaaatttaattttgcagcagtggacg |
| Q439R | tgaaggacgtgatcttgctcgggaaggtaatgaaattattc |
| K94E | catcgagcgtgttgttggagaagaggatcaatatattgcttatgtagc |
| V474T | tatttaattttgcagcagtggacactttggataagtaatcgaccaggc |
| E19D | ggattcaaagctggtgttaaagattacaaattgacttattatactcc |
| Q356K | tgatttactgcgtgatgattttgttgaaaaagatcgaagtcgcg |
| Q30E | tgacttattatactcctgagtacgagaccaaggatactgatatattggc |
| K429Q | agtagctctagaagcatgtgtacaggctcgtaatgaaggacg |
| N468E | cttgtgaagtatggaaagagatcgtatttgagtttgcagcagtggacg |
| V91P | tgctaccgcatcgagcgtgttcctggagaaaaagatcaata |
| A369V | ttatttcactcaagattgggtctctttaccaggtgttctac |
| gg-1 | aaggtctccaaacagagactaaagctagcg |
| gg-2 | aagaggtctccttatccaaaacgtccact |
| gg-3 | aaggtctcgataagtaatcgacc |
| gg-4 | aagaggtctctgtttgtggtgacat |

**Table S8.** Plasmid sequences (see Excel file)**.**

## SUPPLEMENTARY EQUATIONS

$$A_c = \frac{B(C_c.s_c - 0.5O/S_{\mathrm{C/O}})k_{cat}^{C}}{C_c.s_c + K_{\mathrm{C}}^{air}} - R_d$$

**Equation S1.** $A_c$; Rubisco activity-limited photosynthesis rate (*1, 58, 90*). *B*; Rubisco catalytic sites per leaf area (20 μmol sites $m^{-2}$). $C_c$; mesophyll chloroplast $CO_2$ partial pressure (µbar). $s_c$; $CO_2$ solubility in water (0.0334 $M.bar^{-1}$ equivalent to 0.0334 $\mu M.\mu bar^{-1}$). *O*; dissolved stromal $O_2$ concentration (253 μM). $S_{C/O}$; Rubisco specificity factor. $k_{cat}^{CO_2}$; carboxylation rate ($s^{-1}$). $K_{\mathrm{C}}^{air}$; (μM) the apparent Michaelis constant for $CO_2$ at ambient (21% [v/v]) $O_2$. $R_d$; non-photorespiratory $CO_2$ production rate (1 $\mu M.m^{-2}.s^{-1}$). $K_{\mathrm{C}}^{air}$ (i.e. $K_{\mathrm{C}}^{21\%\mathrm{O2}}$) is equivalent to $K_C(1+O/K_O)$, as used previously (*44*).

$$A_j = \frac{(C_c.s_c - 0.5O/S_{\mathrm{C/O}})\,J}{4(C_c.s_c + O/S_{\mathrm{C/O}})} - R_d$$

**Equation S2.** $A_j$, electron transport-limited photosynthesis rate (*1, 58, 90*). Variables described in equation S1. *J*; electron transport rate (120 $\mu mol.m^{-2}.s^{-1}$).

## SUPPLEMENTARY FIGURES

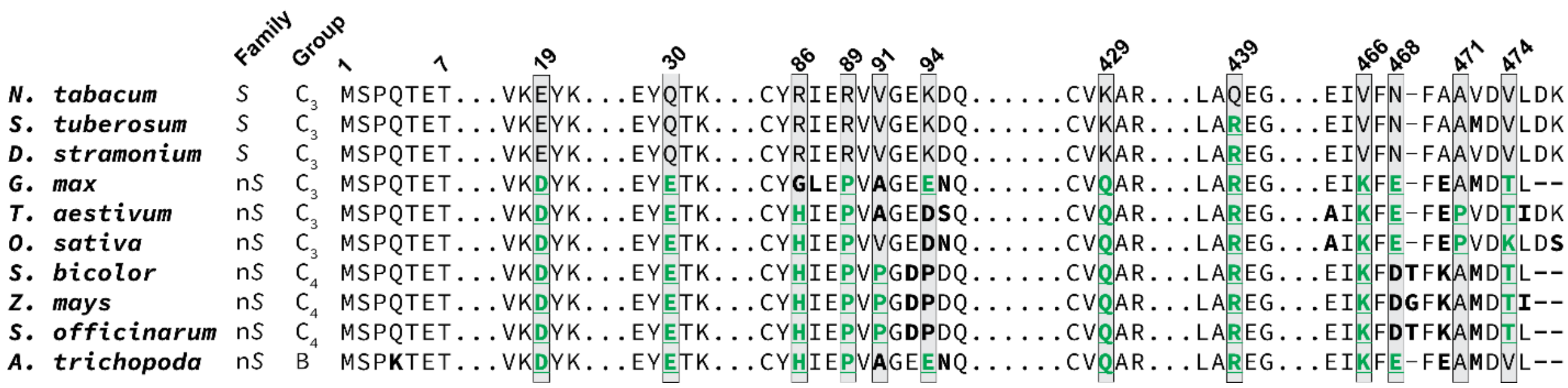


**Figure S1. Alignment of tobacco and other plant RbcL sequences.** Sites of ESM-1bv recommended variants are boxed and recommended amino acid changes are underlined in green. Sequences were aligned using MAFFT (*81*) under default settings. All sequence differences from tobacco Rubisco are bolded. *S*; *Solanaceae*, n*S*; non-*Solanaceae*, B; basal. Full genus names and GenBank Accession numbers are listed in table S2**.**

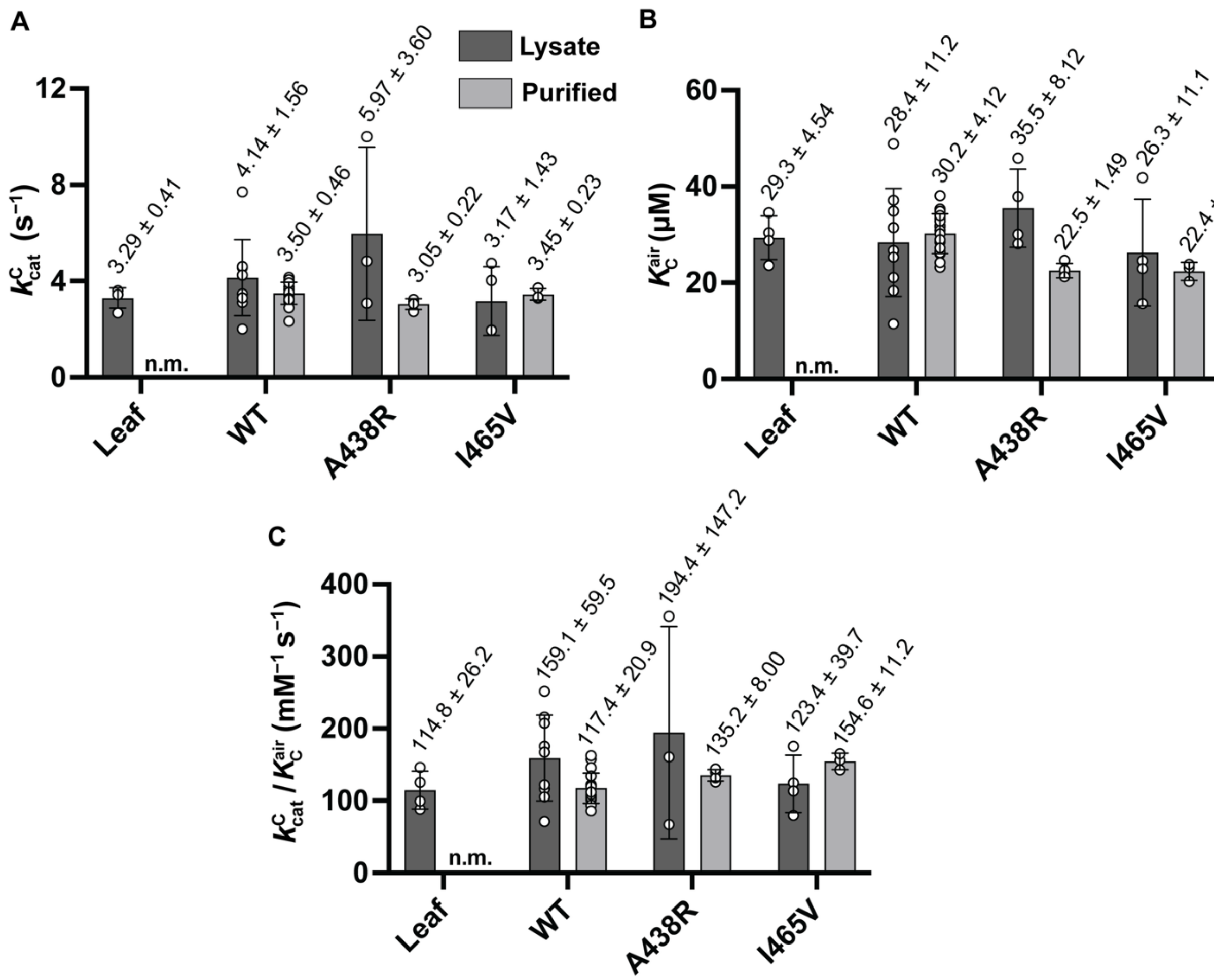


**Figure S2. Kinetic measurements for wild-type *Nt*RbcL, *Nt*RbcL$^{A438R}$, and *Nt*RbcL$^{I465V}$ from *E. coli* lysate and purified protein. A.** Carboxylation rate; $k_{cat}^{C}$. **B.** Affinity for $CO_2$ in air; $K_{C}^{air}$. **C.** Carboxylation efficiency in air; $k_{cat}^{C}/K_{C}^{air}$. Rubisco from *N. tabacum* leaf lysate was included as a positive control in **A**–**C**. Values shown above bars represent mean ± standard deviation. All differences in kinetics between lysate and purified samples had *p*-values > 0.05 when analyzed via two-tailed, heteroscedastic *t*-test.

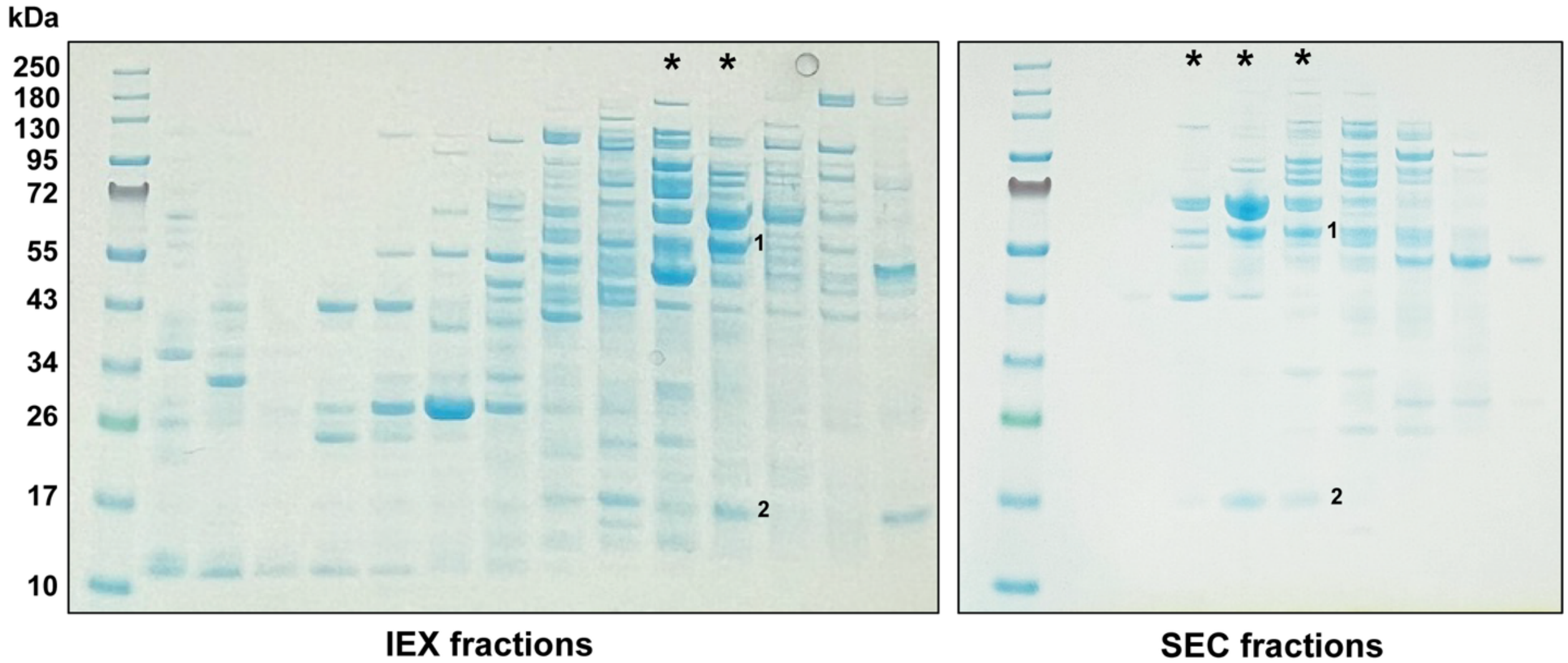


**Figure S3. Purification of untagged tobacco Rubisco from *E. coli* lysate.** Coomassie-stained SDS-PAGE gels show fractions collected after ion exchange (IEX) and size exclusion chromatography (SEC). Fractions containing Rubisco protein are indicated with asterisks. *Nt*RbcL (1) has a molecular weight of 53 kDa and *Nt*RbcS (2) has a molecular weight of 15 kDa.

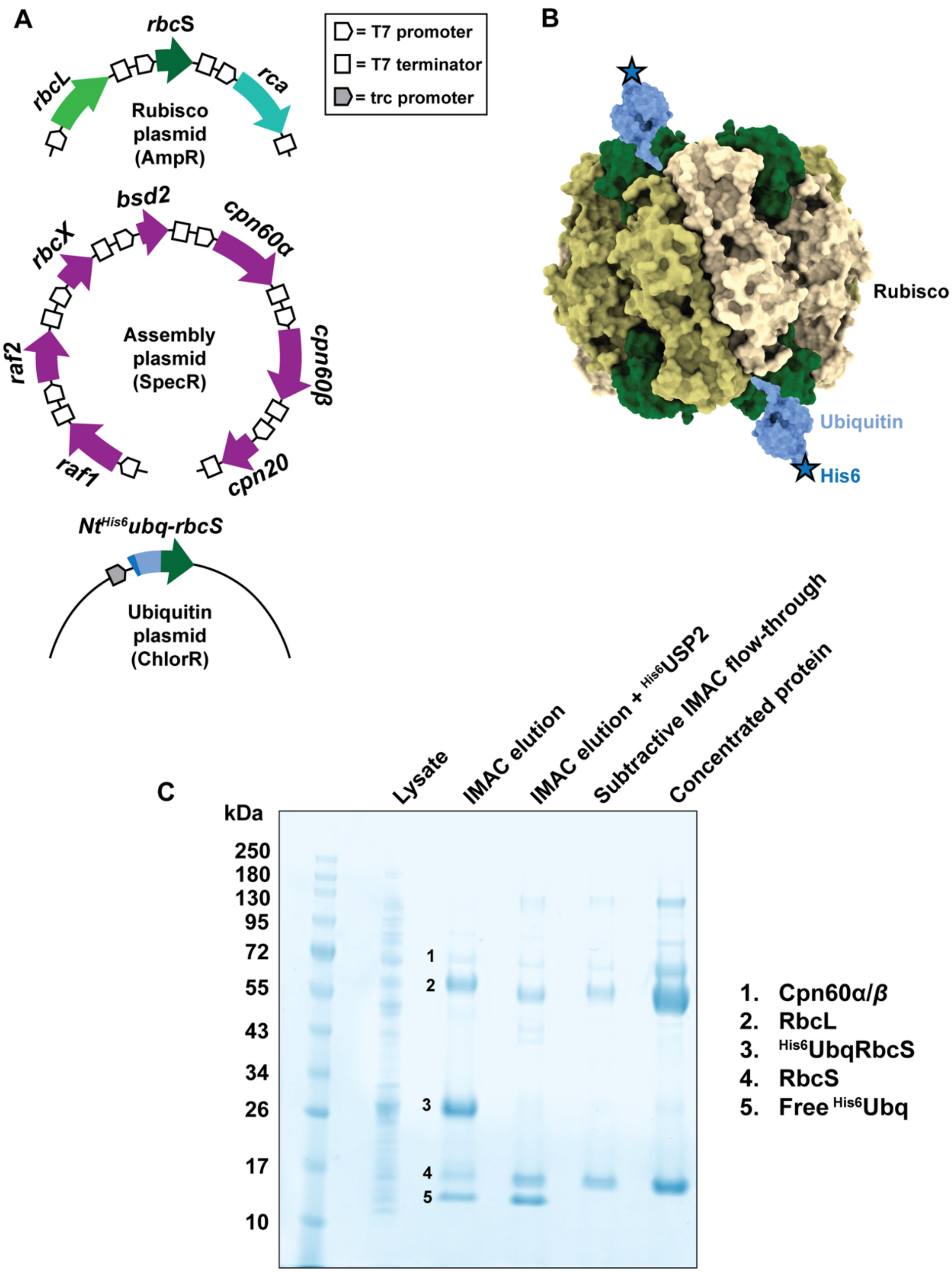


**Figure S4. Histidine-ubiquitin-tagged purification of *N. tabacum* Rubisco complexes. A**. Three-plasmid system for tagged Rubisco expression. Rubisco and assembly plasmids are from Buck *et al* (*24*). **B.** Hypothetical structure of $^{His6}$Ubq-tagged Rubisco. Rubisco holoenzymes are fused to $^{His6}$Ubq at the N-terminus of RbcS, enabling pull-down via nickel immobilized metal affinity chromatography (IMAC). Ubiquitin (PDBID: 3U30 (*91*)) was docked onto the tobacco Rubisco structure (PDBID: 4RUB (*50*)) using ChimeraX (*88*). **C**. Purification of wild-type tobacco Rubisco via histidine-ubiquitin tag.

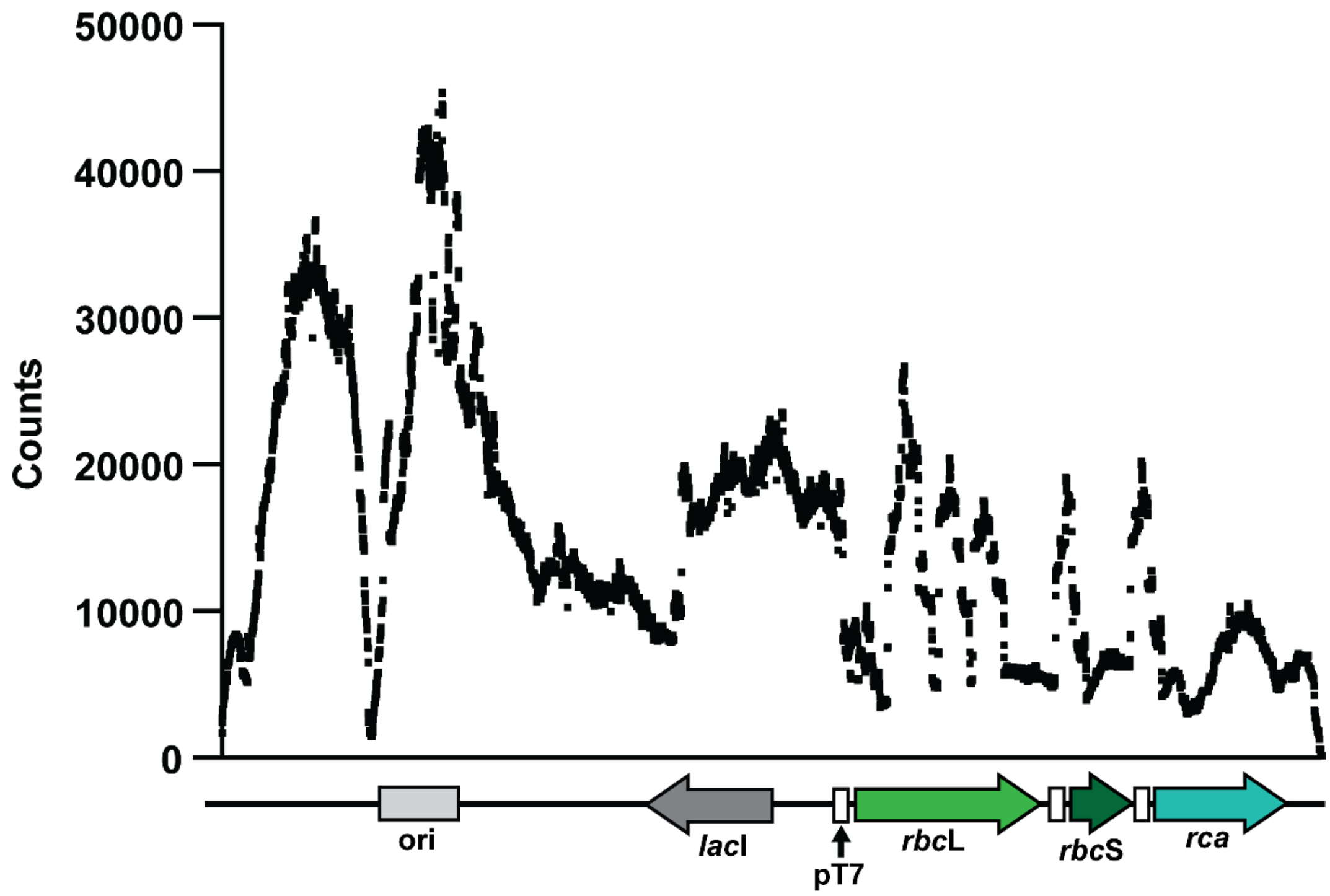


**Figure S5. Sequencing depth across pET-*Nt*LS-Rca.** Data represent the sum of six total samples: technical duplicates of three independent library preparations. Short-read DNA sequencing was performed using a 150 paired-end kit on an Element Biosciences AVITI™ sequencer, as previously described (*11*).

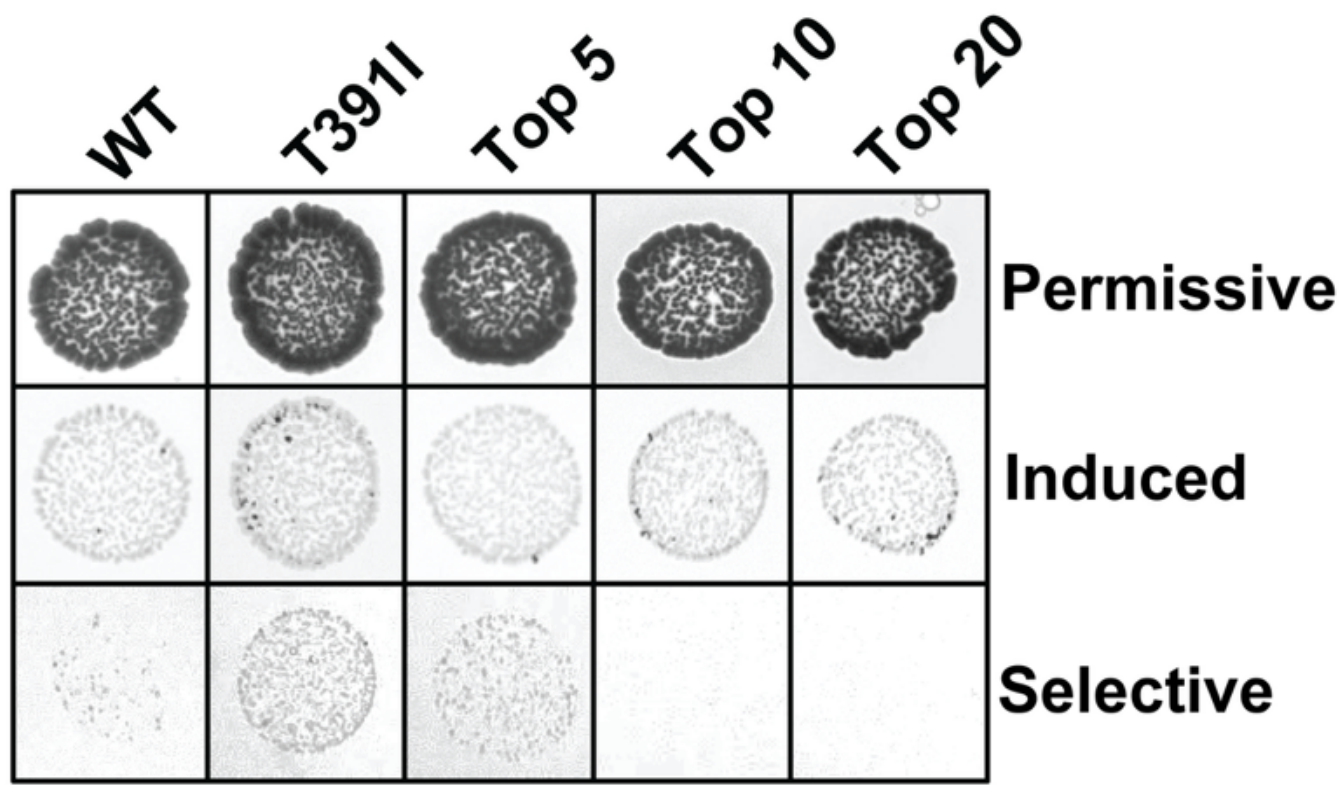


**Top 5:** *Nt*RbcL T391I, I465V, A470P, A369T, N468Q
**Top 10:** Top 5 + R446M, Q30P, V91M, W451Q, I251D
**Top 20:** Top 10 + T23R, V472A, E19D, K94Q, D340N, Q439G, N442P, E464R, V466F, Q356M

**Figure S6. Growth of combinatorial *Nt*RbcL variants in RDE2.** Cells were spot-tested on permissive media containing no additives, induced media containing 0.2 mM IPTG for Rubisco expression, and selective media containing 0.15% [w/v] L-arabinose, 400 μg/mL kanamycin and 0.2 mM IPTG for inducing both PRK and Rubisco expression.

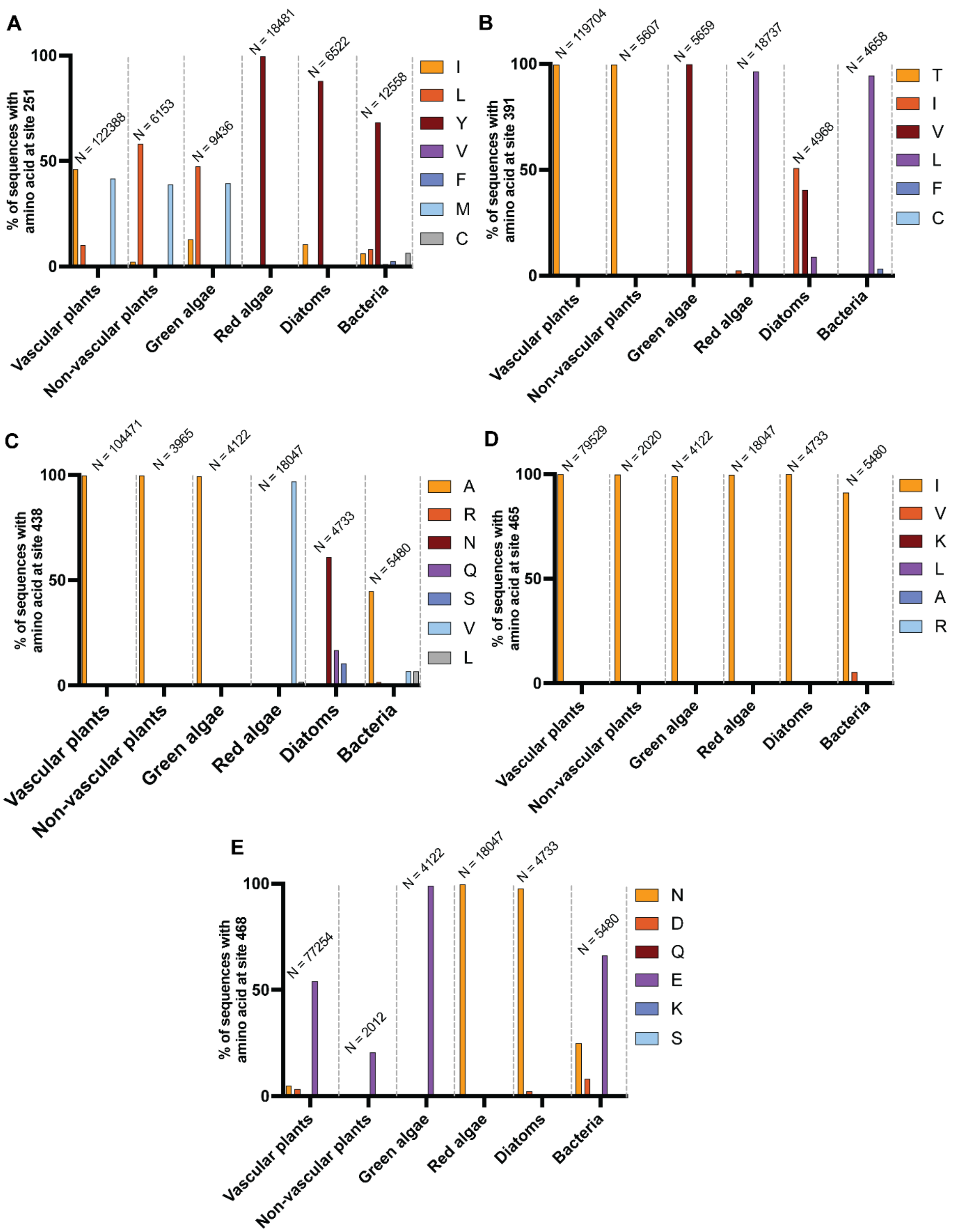


**Figure S7. Phylogenetic analysis at position of selected residues.** Data are shown as a percentage of the total sequences containing the indicated residue at that position. *N* is equal to the number of sequences that had any amino acid residue at the position; thus partial sequences with missing termini are not represented. **A.** Site 251. **B.** Site 391. **C.** Site 438. **D.** Site 465. **E.** Site 468.

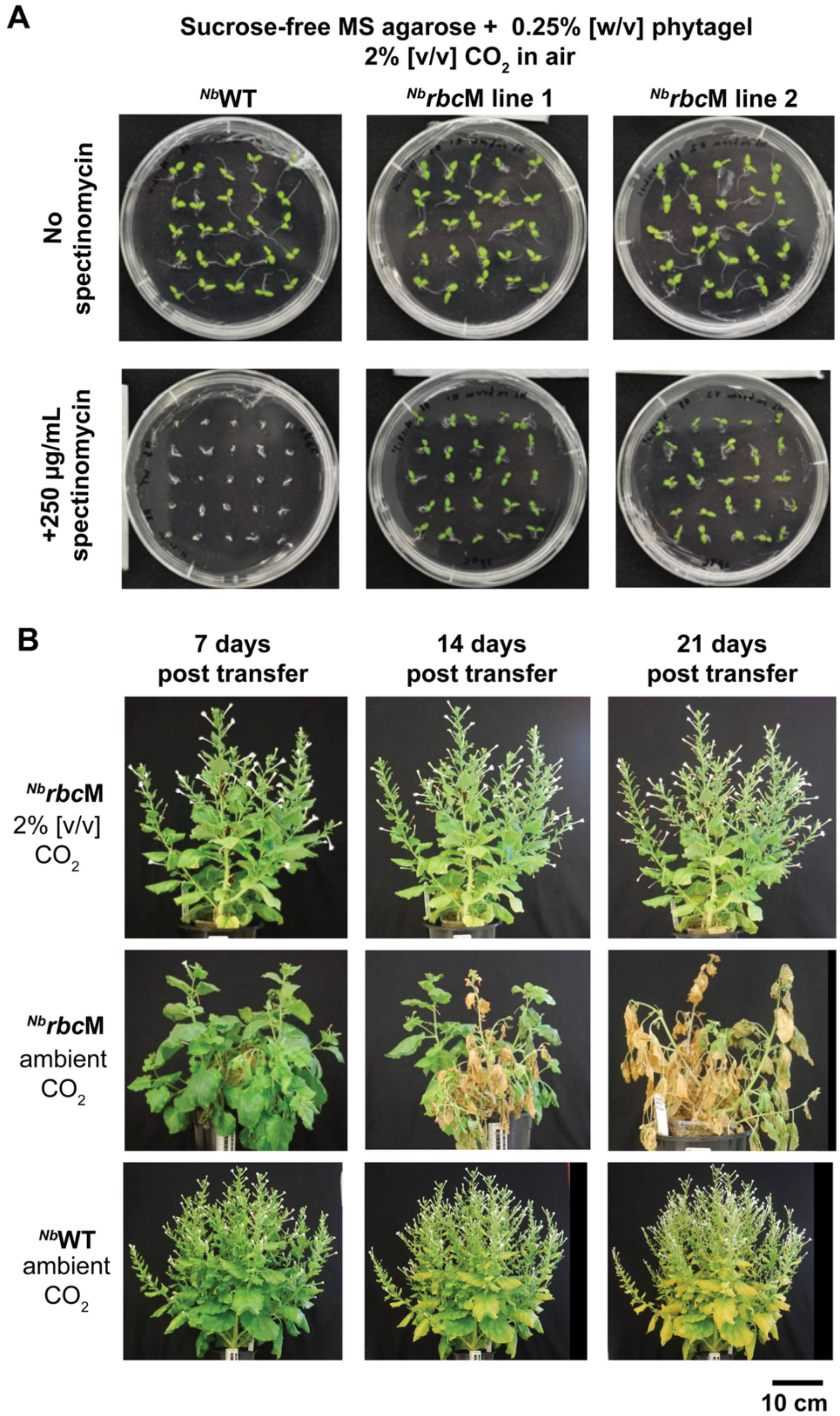


**Figure S8. Germination and growth of $^{Nb}$*rbc*M under elevated $CO_2$. A.** Germination of *N. benthamiana* wild-type ($^{Nb}$WT) and $T_1$ $^{Nb}$*rbc*M plants on media containing spectinomycin shows homoplastomic $^{cm}$*rbc*M-*aad*A integration. **B.** The $T_1$ plants were propagated in soil for 32 days under elevated $CO_2$ (air + 2% [v/v] $CO_2$) and then either maintained under elevated $CO_2$ or grown with identical chamber conditions under ambient $CO_2$ (air + 0.06% [v/v] $CO_2$) for 7–21 days and imaged. Vegetative growth and seed setting by the $^{Nb}$*rbc*M lines was supported under elevated $CO_2$, but not ambient.

## REFERENCES

1. M. Gionfriddo, T. Rhodes, S. M. Whitney, Perspectives on improving crop Rubisco by directed evolution. *Semin. Cell Dev. Biol.* **155**, 37–47 (2024).

2. R. E. Sharwood, Engineering chloroplasts to improve Rubisco catalysis: Prospects for translating improvements into food and fiber crops. *New Phytol.* **213**, 494–510 (2017).

3. N. Prywes, N. R. Phillips, O. T. Tuck, L. E. Valentin-Alvarado, D. F. Savage, Rubisco function, evolution, and engineering. *Annu. Rev. Biochem.* **92**, 385–410 (2023).

4. E. Carmo-Silva, J. C. Scales, P. J. Madgwick, M. A. J. Parry, Optimizing Rubisco and its regulation for greater resource use efficiency. *Plant Cell Environ.* **38**, 1817-1832 (2014).

5. B. J. Walker, A. VanLoocke, C. J. Bernacchi, D. R. Ort, The costs of photorespiration to food production now and in the future. *Annu. Rev. Plant Biol.* **67**, 107–129 (2016).

6. G. G. B. Tcherkez, G. D. Farquhar, T. J. Andrews, Despite slow catalysis and confused substrate specificity, all ribulose bisphosphate carboxylases may be nearly perfectly optimized. *Proc. Natl. Acad. Sci. U.S.A.* **103**, 7246–7251 (2006).

7. J. Galmés, M. V. Kapralov, P. J. Andralojc, M. À. Conesa, A. J. Keys, M. A. J. Parry, J. Flexas, Expanding knowledge of the Rubisco kinetics variability in plant species: Environmental and evolutionary trends. *Plant Cell Environ.* **37**, 1989–2001 (2014).

8. P. L. Cummins, B. Kannappan, J. E. Gready, Directions for optimization of photosynthetic carbon fixation: Rubisco's efficiency may not be so constrained after all. *Front. Plant Sci.* **9**, 183 (2018).

9. J. W. Bouvier, D. M. Emms, S. Kelly, Rubisco is evolving for improved catalytic efficiency and $CO_2$ assimilation in plants. *Proc. Natl. Acad. Sci. U.S.A.* **121**, e2321050121 (2024).

10. J. W. Bouvier, D. M. Emms, T. Rhodes, J. S. Bolton, A. Brasnett, A. Eddershaw, J. R. Nielsen, A. Unitt, S. M. Whitney, S. Kelly, Rubisco adaptation is more limited by phylogenetic constraint than by catalytic trade-off. *Mol. Biol. Evol.* **38**, 2880–2896 (2021).

11. J. L. McDonald, N. P. Shapiro, A. A. Mengiste, S. Kaines, S. M. Whitney, R. H. Wilson, M. D. Shoulders, *In vivo* directed evolution of an ultrafast Rubisco from a semianaerobic environment imparts oxygen resistance. *Proc. Natl. Acad. Sci. U.S.A.* **122**, e2505083122 (2025).

12. M. Gionfriddo, R. Birch, T. Rhodes, S. Buck, T. Skinner, I. Andersson, S. Whitney, Laboratory evolution of Rubisco solubility and catalytic switches to enhance plant productivity. *Nat. Plants* **11**, 1939–1950 (2025).

13. R. H. Wilson, H. Alonso, S. M. Whitney, Evolving *Methanococcoides burtonii* archaeal Rubisco for improved photosynthesis and plant growth. *Sci. Rep.* **6**, 22284 (2016).

14. R. H. Wilson, E. Martin-Avila, C. Conlan, S. M. Whitney, An improved *Escherichia coli* screen for rubisco identifies a protein–protein interface that can enhance $CO_2$-fixation kinetics. *J. Biol. Chem.* **293**, 18–27 (2018).

15. N. Prywes, N. R. Phillips, L. M. Oltrogge, S. Lindner, L. J. Taylor-Kearney, Y.-C. C. Tsai, B. de Pins, A. E. Cowan, H. A. Chang, R. Z. Wang, L. N. Hall, D. Bellieny-Rabelo, H. M. Nisonoff, R. F. Weissman, A. I. Flamholz, D. Ding, A. Y. Bhatt, O. Mueller-Cajar, P. M. Shih, R. Milo, D. F. Savage, A map of the rubisco biochemical landscape. *Nature* **638**, 823–828 (2025).

16. G. Bainbridge, P. Madgwick, S. Parmar, R. Mitchell, M. Paul, J. Pitts, A. J. Keys, M. A. J. Parry, Engineering Rubisco to change its catalytic properties. *J. Exp. Bot.* **46**, 1269–1276 (1995).

17. M. R. Badger, R. E. Sharwood, Rubisco, the imperfect winner: It's all about the base. *J. Exp. Bot.* **74**, 562–580 (2023).

18. L. Schulz, J. Zarzycki, W. Steinchen, G. K. A. Hochberg, T. J. Erb, Layered entrenchment maintains essentiality in the evolution of Form I Rubisco complexes. *EMBO J.* **44**, 269–280 (2025).

19. Z. G. Oh, B. Askey, L. H. Gunn, Red Rubiscos and opportunities for engineering green plants. *J. Exp. Bot.* **74**, 520–542 (2023).

20. H. Aigner, R. H. Wilson, A. Bracher, L. Calisse, J. Y. Bhat, F. U. Hartl, M. Hayer-Hartl, Plant rubisco assembly in *E. coli* with five chloroplast chaperones including BSD2. *Science* **358**, 1272–1278 (2017).

21. R. H. Wilson, G. Thieulin-Pardo, F. U. Hartl, M. Hayer-Hartl, Improved recombinant expression and purification of functional plant rubisco. *FEBS Lett.* **593**, 611–621 (2019).

22. M. T. Lin, H. Salihovic, F. K. Clark, M. R. Hanson, Improving the efficiency of Rubisco by resurrecting its ancestors in the family *Solanaceae*. *Sci. Adv.* **8**, eabm6871 (2022).

23. M. T. Lin, W. D. Stone, V. Chaudhari, M. R. Hanson, Small subunits can determine enzyme kinetics of tobacco rubisco expressed in *Escherichia coli*. *Nat. Plants* **6**, 1289–1299 (2020).

24. S. Buck, T. Rhodes, M. Gionfriddo, T. Skinner, D. Yuan, R. Birch, M. V. Kapralov, S. M. Whitney, *Escherichia coli* expressing chloroplast chaperones as a proxy to test heterologous Rubisco production in leaves. *J. Exp. Bot.* **74**, 664–676 (2023).

25. Z. Cai, L. Zhao, K. Ma, G. Liu, J. Zhang, F. Gong, W. Pei, L. Xiao, Y. Li, Replacement of large subunit N terminus enabled biogenesis of different plant Rubiscos in *E. coli*. *Plant Biotech. J.* **23**, 3382–3391 (2025).

26. J. Archer, M. Kathpalia, B. U. Lee, S. Li, T. Wang, Effects of chaperone selectivity on the assembly of plant Rubisco orthologs in *E. coli*. *J. Exp. Bot.* **76**, 2809–2820 (2025).

27. Z. G. Oh, T. A. Robison, D. H. Loh, W. S. L. Ang, J. Z. Y. Ng, F.-W. Li, L. H. Gunn, Unique biogenesis and kinetics of hornwort Rubiscos revealed by synthetic biology systems. *Mol. Plant* **17**, 1833–1849 (2024).

28. Y. Zhou, S. Whitney, Directed evolution of an improved rubisco; *In vitro* analyses to decipher fact from fiction. *Int. J. Mol. Sci.* **20**, e5019 (2019).

29. P. Kouba, P. Kohout, F. Haddadi, A. Bushuiev, R. Samusevich, J. Sedlar, J. Damborsky, T. Pluskal, J. Sivic, S. Mazurenko, Machine learning-guided protein engineering. *ACS Catal.* **13**, 13863–13895 (2023).

30. S. Mazurenko, Z. Prokop, J. Damborsky, Machine learning in enzyme engineering. *ACS Catal.* **10**, 1210–1223 (2019).

31. Y. Qiu, G.-W. Wei, Artificial intelligence-aided protein engineering: From topological data analysis to deep protein language models. *Brief. Bioinform.* **24**, bbad289 (2023).

32. E. Simon, K. Swanson, J. Zou, Language models for biological research: A primer. *Nat. Methods* **21**, 1422–1429 (2024).

33. J. Yang, F.-Z. Li, F. H. Arnold, Opportunities and challenges for machine learning-assisted enzyme engineering. *ACS Cent. Sci.* **10**, 226–241 (2024).

34. B. L. Hie, V. R. Shanker, D. Xu, T. U. J. Bruun, P. A. Weidenbacher, S. Tang, W. Wu, J. E. Pak, P. S. Kim, Efficient evolution of human antibodies from general protein language models. *Nat. Biotechnol.* **42**, 275–283 (2024).

35. V. R. Shanker, T. U. J. Bruun, B. L. Hie, P. S. Kim, Unsupervised evolution of protein and antibody complexes with a structure-informed language model. *Science* **385**, 46–53 (2024).

36. N. Thomas, D. Belanger, C. Xu, H. Lee, K. Hirano, K. Iwai, V. Polic, K. D. Nyberg, K. G. Hoff, L. Frenz, C. A. Emrich, J. W. Kim, M. Chavarha, A. Ramanan, J. J. Agresti, L. J. Colwell, Engineering highly active nuclease enzymes with machine learning and high-throughput screening. *Cell Syst.* **16**, 101236 (2025).

37. B. J. Wittmann, Y. Yue, F. H. Arnold, Informed training set design enables efficient machine learning-assisted directed protein evolution. *Cell Syst.* **12**, 1026–1045.e7 (2021).

38. Z. Lin, H. Akin, R. Rao, B. Hie, Z. Zhu, W. Lu, N. Smetanin, R. Verkuil, O. Kabeli, Y. Shmueli, A. D. S. Costa, M. Fazel-Zarandi, T. Sercu, S. Candido, A. Rives, Evolutionary-scale prediction of atomic-level protein structure with a language model. *Science* **379**, 1123–1130 (2023).

39. K. Jiang, Z. Yan, M. D. Bernardo, S. R. Sgrizzi, L. Villiger, A. Kayabolen, B. J. Kim, J. K. Carscadden, M. Hiraizumi, H. Nishimasu, J. S. Gootenberg, O. O. Abudayyeh, Rapid *in silico* directed evolution by a protein language model with EVOLVEpro. *Science* **387**, eadr6006 (2025).

40. W. A. Iqbal, A. Lisitsa, M. V. Kapralov, Predicting plant Rubisco kinetics from RbcL sequence data using machine learning. *J. Exp. Bot.* **74**, 638–650 (2023).

41. J. Meier, R. Rao, R. Verkuil, J. Liu, T. Sercu, A. Rives, Language models enable zero-shot prediction of the effects of mutations on protein function. *Adv. Neural Inf. Proces. Syst.* **34**, 29287–29303 (2021).

42. A. Rives, J. Meier, T. Sercu, S. Goyal, Z. Lin, J. Liu, D. Guo, M. Ott, C. L. Zitnick, J. Ma, R. Fergus, Biological structure and function emerge from scaling unsupervised learning to 250 million protein sequences. *Proc. Natl. Acad. Sci. U.S.A.* **118**, e2016239118 (2021).

43. D. J. Orr, A. Alcântara, M. V. Kapralov, P. J. Andralojc, E. Carmo-Silva, M. A. J. Parry, Surveying Rubisco diversity and temperature response to improve crop photosynthetic efficiency. *Plant Physiol.* **172**, 707–717 (2016).

44. R. E. Sharwood, O. Ghannoum, M. V. Kapralov, L. H. Gunn, S. M. Whitney, Temperature responses of Rubisco from *Paniceae* grasses provide opportunities for improving $C_3$ photosynthesis. *Nat. Plants* **2**, 16186 (2016).

45. J. Galmés, P. J. Andralojc, M. V. Kapralov, J. Flexas, A. J. Keys, A. Molins, M. A. J. Parry, M. À. Conesa, Environmentally driven evolution of Rubisco and improved photosynthesis and growth within the $C_3$ genus *Limonium* (*P. lumbaginaceae*). *New Phytol.* **203**, 989–999 (2014).

46. C. Hsu, R. Verkuil, J. Liu, Z. Lin, B. Hie, T. Sercu, A. Lerer, A. Rives, in *International Conference on Machine Learning* (PMLR, 2022), pp. 8946–8970.

47. J. Jumper, R. Evans, A. Pritzel, T. Green, M. Figurnov, O. Ronneberger, K. Tunyasuvunakool, R. Bates, A. Žídek, A. Potapenko, A. Bridgland, C. Meyer, S. A. A. Kohl, A. J. Ballard, A. Cowie, B. Romera-Paredes, S. Nikolov, R. Jain, J. Adler, T. Back, S. Petersen, D. Reiman, E.

Clancy, M. Zielinski, M. Steinegger, M. Pacholska, T. Berghammer, S. Bodenstein, D. Silver, O. Vinyals, A. W. Senior, K. Kavukcuoglu, P. Kohli, D. Hassabis, Highly accurate protein structure prediction with AlphaFold. *Nature* **596**, 583–589 (2021).

48. C. Hsu, H. Nisonoff, C. Fannjiang, J. Listgarten, Learning protein fitness models from evolutionary and assay-labeled data. *Nat. Biotech.* **40**, 1114–1122 (2022).

49. K. Y. J. Zhang, D. Cascio, D. Eisenberg, Crystal structure of the unactivated ribulose 1, 5-bisphosphate carboxylase/oxygenase complexed with a transition state analog, 2-carboxy-D-arabinitol 1, 5-bisphosphate. *Protein Sci.* **3**, 64–69 (1994).

50. S. W. Suh, D. Cascio, M. S. Chapman, D. Eisenberg, A crystal form of ribulose-1,5-bisphosphate carboxylase/oxygenase from *Nicotiana tabacum* in the activated state. *J. Mol. Biol.* **197**, 363–365 (1987).

51. T. C. Taylor, I. Andersson, Structural transitions during activation and ligand binding in hexadecameric Rubisco inferred from the crystal structure of the activated unliganded spinach enzyme. *Nat. Struct. Mol. Biol.* **3**, 95–101 (1996).

52. O. Mueller-Cajar, S. M. Whitney, Evolving improved *Synechococcus* Rubisco functional expression in *Escherichia coli*. *Biochem. J.* **414**, 205–214 (2008).

53. P. Durão, H. Aigner, P. Nagy, O. Mueller-Cajar, F. U. Hartl, M. Hayer-Hartl, Opposing effects of folding and assembly chaperones on evolvability of Rubisco. *Nat. Chem. Biol.* **11**, 148–155 (2015).

54. M. R. Parikh, D. N. Greene, K. K. Woods, I. Matsumura, Directed evolution of RuBisCO hypermorphs through genetic selection in engineered *E. coli*. *Protein Eng. Des. Sel.* **19**, 113–119 (2006).

55. J. Amaral, A. K. M. Lobo, E. Carmo-Silva, D. J. Orr, "Purification of Rubisco from leaves" in *Photosynthesis: Methods and Protocols*, S. Covshoff, Ed. (Springer US, 2024), pp. 417–426.

56. R. T. Baker, A. M. Catanzariti, Y. Karunasekara, T. A. Soboleva, R. Sharwood, S. Whitney, P. G. Board, Using deubiquitylating enzymes as research tools. *Methods Enzymol.* **398**, 540–554 (2005).

57. H. M. Steiger, E. Beck, R. Beck, Oxygen concentration in isolated chloroplasts during photosynthesis. *Plant Physiol.* **60**, 903–906 (1977).

58. G. D. Farquhar, S. von Caemmerer, J. A. Berry, A biochemical model of photosynthetic $CO_2$ assimilation in leaves of $C_3$ species. *Planta* **149**, 78–90 (1980).

59. A. I. Flamholz, N. Prywes, U. Moran, D. Davidi, Y. M. Bar-On, L. M. Oltrogge, R. Alves, D. Savage, R. Milo, Revisiting trade-offs between rubisco kinetic parameters. *Biochemistry* **58**, 3365–3376 (2019).

60. Y. Savir, E. Noor, R. Milo, T. Tlusty, Cross-species analysis traces adaptation of rubisco toward optimality in a low-dimensional landscape. *Proc. Natl. Acad. Sci. U.S.A.* **107**, 3475–3480 (2010).

61. S. Satagopan, R. J. Spreitzer, Substitutions at the Asp-473 latch residue of *Chlamydomonas* Ribulose bisphosphate carboxylase/oxygenase cause decreases in carboxylation efficiency and $CO_2/O_2$ specificity. *J. Biol. Chem.* **279**, 14240–14244 (2004).

62. S. Gutteridge, J. Newman, C. Herrmann, D. Rhoades, The crystal structures of Rubisco and opportunities for manipulating photosynthesis. *J. Exp. Bot.* **46**, 1261–1267 (1995).

63. A. Bracher, A. Starling-Windhof, F. U. Hartl, M. Hayer-Hartl, Crystal structure of a chaperone-bound assembly intermediate of form I Rubisco. *Nat. Struct. Mol. Biol.* **18**, 875–880 (2011).

64. M. V. Kapralov, D. A. Filatov, Widespread positive selection in the photosynthetic Rubisco enzyme. *BMC Evol. Biol.* **7**, 73 (2007).

65. B. Stec, Structural mechanism of RuBisCO activation by carbamylation of the active site lysine. *Proc. Natl. Acad. Sci. U.S.A.* **109**, 18785–18790 (2012).

66. S. M. Whitney, R. E. Sharwood, Construction of a tobacco master line to improve Rubisco engineering in chloroplasts. *J. Exp. Bot.* **59**, 1909–1921 (2008).

67. T. Manning, R. Birch, T. Stevenson, G. Nugent, S. Whitney, Bacterial Form II Rubisco can support wild-type growth and productivity in *Solanum tuberosum* cv. Desiree (potato) under elevated $CO_2$. *PNAS Nexus* **2**, pgac305 (2023).

68. Y. Zhou, L. H. Gunn, R. Birch, I. Andersson, S. M. Whitney, Grafting *Rhodobacter sphaeroides* with red algae Rubisco to accelerate catalysis and plant growth. *Nat. Plants* **9**, 978–986 (2023).

69. S. M. Whitney, S. von Caemmerer, G. S. Hudson, T. J. Andrews, Directed mutation of the Rubisco large subunit of tobacco influences photorespiration and growth. *Plant Physiol.* **121**, 579–588 (1999).

70. M. T. Lin, A. Occhialini, P. J. Andralojc, M. A. J. Parry, M. R. Hanson, A faster Rubisco with potential to increase photosynthesis in crops. *Nature* **513**, 547–550 (2014).

71. A. Occhialini, M. T. Lin, P. J. Andralojc, M. R. Hanson, M. A. J. Parry, Transgenic tobacco plants with improved cyanobacterial Rubisco expression but no extra assembly factors grow at near wild-type rates if provided with elevated $CO_2$. *Plant J.* **85**, 148–160 (2016).

72. J. Ng, Z. Guo, O. Mueller-Cajar, Rubisco activase requires residues in the large subunit N terminus to remodel inhibited plant Rubisco. *J. Biol. Chem.* **295**, 16427–16435 (2020).

73. E. M. Larson, C. M. O'Brien, G. Zhu, R. J. Spreitzer, A. R. Portis, Specificity for activase is changed by a Pro-89 to Arg substitution in the large subunit of ribulose-1,5-bisphosphate carboxylase/oxygenase. *J. Biol. Chem.* **272**, 17033–17037 (1997).

74. C. M. Ott, B. D. Smith, A. R. Portis Jr., R. J. Spreitzer, Activase region on chloroplast ribulose-1,5-bisphosphate carboxylase/oxygenase: Nonconservative substitution in the large subunit alters species specificity of protein interaction. *J. Biol. Chem.* **275**, 26241–26244 (2000).

75. L. H. Gunn, E. Martin Avila, R. Birch, S. M. Whitney, The dependency of red Rubisco on its cognate activase for enhancing plant photosynthesis and growth. *Proc. Natl. Acad. Sci. U.S.A.* **117**, 25890–25896 (2020).

76. S. M. Whitney, R. Birch, C. Kelso, J. L. Beck, M. V. Kapralov, Improving recombinant Rubisco biogenesis, plant photosynthesis and growth by coexpressing its ancillary RAF1 chaperone. *Proc. Natl. Acad. Sci. U.S.A.* **112**, 3564–3569 (2015).

77. A. Prins, D. J. Orr, P. J. Andralojc, M. P. Reynolds, E. Carmo-Silva, M. A. J. Parry, Rubisco catalytic properties of wild and domesticated relatives provide scope for improving wheat photosynthesis. *J. Exp. Bot.* **67**, 1827–1838 (2016).

78. M. T. Lin, D. J. Orr, D. Worrall, M. A. J. Parry, E. Carmo-Silva, M. R. Hanson, A procedure to introduce point mutations into the Rubisco large subunit gene in wild-type plants. *Plant J.* **106**, 876–887 (2021).

79. R. Flachmann, G. Zhu, R. G. Jensen, H. J. Bohnert, Mutations in the small subunit of ribulose-1,5-bisphosphate carboxylase/oxygenase increase the formation of the misfire product xylulose-1,5-bisphosphate. *Plant Physiol.* **114**, 131–136 (1997).

80. L. Schulz, Z. Guo, J. Zarzycki, W. Steinchen, J. M. Schuller, T. Heimerl, S. Prinz, O. Mueller-Cajar, T. J. Erb, G. K. A. Hochberg, Evolution of increased complexity and specificity at the dawn of form I Rubiscos. *Science* **378**, 155–160 (2022).

81. K. Katoh, D. M. Standley, MAFFT multiple sequence alignment software version 7: Improvements in performance and usability. *Mol. Biol. Evol.* **30**, 772–780 (2013).

82. L. S. Johnson, S. R. Eddy, E. Portugaly, Hidden Markov model speed heuristic and iterative HMM search procedure. *BMC Bioinformatics* **11**, 431 (2010).

83. G. A. Van der Auwera, B. D. O'Connor, *Genomics in the Cloud: Using Docker, GATK, and WDL in Terra* (O'Reilly Media Inc., 2020), pp. 496.

84. H. J. Kane, J. Viil, B. Entsch, K. Paul, M. K. Morell, T. J. Andrews, An improved method for measuring the $CO_2/O_2$ specificity of Ribulose bisphosphate Carboxylase-Oxygenase. *Funct. Plant Biol.* **21**, 449 (1994).

85. D. S. Kubien, C. M. Brown, H. J. Kane, Quantifying the amount and activity of Rubisco in leaves. *Methods Mol. Biol.* **684**, 349–362 (2011).

86. S. M. Whitney, R. E. Sharwood, "Plastid transformation for Rubisco engineering and protocols for assessing expression" in *Chloroplast Biotechnology: Methods and Protocols*, Methods in Molecular Biology (Humana Press, 2014), pp. 245–262.

87. S. M. Whitney, H. J. Kane, R. L. Houtz, R. E. Sharwood, Rubisco oligomers composed of linked small and large subunits assemble in tobacco plastids and have higher affinities for $CO_2$ and $O_2$. *Plant Physiol.* **149**, 1887–1895 (2009).

88. A. P. Duff, T. J. Andrews, P. M. G. Curmi, The transition between the open and closed states of rubisco is triggered by the inter-phosphate distance of the bound bisphosphate. *J. Mol. Biol.* **298**, 903–916 (2000).

89. E. C. Meng, T. D. Goddard, E. F. Pettersen, G. S. Couch, Z. J. Pearson, J. H. Morris, T. E. Ferrin, UCSF ChimeraX: Tools for structure building and analysis. *Protein Sci.* **32**, e4792 (2023).

90. R. E. Sharwood, O. Ghannoum, S. M. Whitney, Prospects for improving $CO_2$ fixation in $C_3$-crops through understanding $C_4$-Rubisco biogenesis and catalytic diversity. *Curr. Opin. Plant Biol.* **31**, 135–142 (2016).

91. M. L. Matsumoto, K. C. Dong, C. Yu, L. Phu, X. Gao, R. N. Hannoush, S. G. Hymowitz, D. S. Kirkpatrick, V. M. Dixit, R. F. Kelley, Engineering and structural characterization of a linear polyubiquitin-specific antibody. *J. Mol. Biol.* **418**, 134–144 (2012).
